# First-in-human immunoPET imaging of COVID-19 convalescent patients using dynamic total-body PET and a CD8-targeted minibody

**DOI:** 10.1126/sciadv.adh7968

**Published:** 2023-10-12

**Authors:** Negar Omidvari, Terry Jones, Pat M. Price, April L. Ferre, Jacqueline Lu, Yasser G. Abdelhafez, Fatma Sen, Stuart H. Cohen, Kristin Schmiedehausen, Ramsey D. Badawi, Barbara L. Shacklett, Ian Wilson, Simon R. Cherry

**Affiliations:** ^1^ Department of Biomedical Engineering, University of California Davis, Davis, CA, USA.; ^2^ Department of Radiology, University of California Davis Medical Center, Sacramento, CA, USA.; ^3^ Department of Surgery and Cancer, Imperial College London, London, UK.; ^4^ Department of Medical Microbiology and Immunology, School of Medicine, University of California Davis, Davis, CA, USA.; ^5^ Radiotherapy and Nuclear Medicine Department, South Egypt Cancer Institute, Assiut University, Assiut, Egypt.; ^6^ Division of Infectious Diseases, Department of Internal Medicine, University of California Davis Medical Center, Sacramento, CA, USA.; ^7^ ImaginAb Inc., Inglewood, CA, USA.

## Abstract

With most of the T cells residing in the tissue, not the blood, developing noninvasive methods for in vivo quantification of their biodistribution and kinetics is important for studying their role in immune response and memory. This study presents the first use of dynamic positron emission tomography (PET) and kinetic modeling for in vivo measurement of CD8^+^ T cell biodistribution in humans. A ^89^Zr-labeled CD8-targeted minibody (^89^Zr-Df-Crefmirlimab) was used with total-body PET in healthy individuals (*N* = 3) and coronavirus disease 2019 (COVID-19) convalescent patients (*N* = 5). Kinetic modeling results aligned with T cell–trafficking effects expected in lymphoid organs. Tissue-to-blood ratios from the first 7 hours of imaging were higher in bone marrow of COVID-19 convalescent patients compared to controls, with an increasing trend between 2 and 6 months after infection, consistent with modeled net influx rates and peripheral blood flow cytometry analysis. These results provide a promising platform for using dynamic PET to study the total-body immune response and memory.

## Teaser

Total-body dynamic imaging of a radiolabeled minibody enables measurements of distribution and kinetics of targeted immune cells.

## INTRODUCTION

Understanding the adaptive immune response to viral infections and subsequent immunological memory is critical for the development of vaccines and therapeutic options. Studying the immune response in humans has been conventionally focused on peripheral blood assays, particularly in longitudinal studies, due to complexity and invasive nature of tissue sampling approaches. However, most of the immune cells involved in the adaptive immune response and immunological memory reside and function in tissue, particularly in lymphoid organs such as the bone marrow, spleen, tonsils, and lymph nodes (*1*, *2*). CD8^+^ T cells are one of the key players in cell-mediated immune response against viral infections, and there has been a growing interest in studying the critical role of CD8^+^ T cell trafficking and preferential residence of CD8^+^ memory T cells in certain niches, such as the bone marrow, in immunological memory (*3*–*6*).

The recent pandemic caused by the severe acute respiratory syndrome coronavirus 2 (SARS-CoV-2) emphasized the need to better understand the role of adaptive immunity in viral infections, and there has been particular interest in the role of T cell response to the coronavirus disease 2019 (COVID-19) (*7*, *8*). Immunological memory to SARS-CoV-2 infection has been extensively characterized in the blood (*9*–*12*). In particular, SARS-CoV-2–specific CD8^+^ memory T cell response appears to persist for at least 8 months in the blood, with a declining trend observed between 1 and 8 months after infection (*9*, *10*). Furthermore, examination of SARS-CoV-2 seropositive organ donors has shown SARS-CoV-2–specific CD8^+^ T cell memory in the bone marrow, spleen, lung, and lymph nodes for up to 6 months after infection (*13*).

A noninvasive method capable of quantifying T cell density and trafficking rates in the tissue at a system level for the whole body could enable longitudinal studies in patients with viral infections and in the healthy populations, leading to better understanding of the adaptive immune response and immunological memory. The case for developing such a method for researching COVID-19 infection would include the acute and recovery phases, pre-existing immunity (*14*), asymptomatic response (*15*), and herd immunity (*16*). These could be extended to studies of susceptibility to COVID-19 in association with age (*17*), genetic factors (*18*), gender (*19*), children (*20*), and obesity (*21*). Being able to noninvasively study T cell involvement in peripheral effects (*22*), long COVID (*23*), vaccine efficacy (*24*), and therapeutic interventions *(**25*) would also suggest potentially fruitful areas for whole-body COVID-19 research.

As a proof of concept for staging such a transformative research strategy, a highly sensitive quantitative in vivo imaging methodology targeting human CD8^+^ cells is described, with a particular interest toward studying the immunobiology of CD8^+^ T cells as the major population of human CD8^+^ cells. For this, the recently developed imaging probe ^89^Zr-Df-Crefmirlimab, also known as ^89^Zr-Df-IAB22M2C, is used with positron emission tomography (PET). IAB22M2C is a biologically inert 80-kDa minibody with high affinity to human CD8 and has accelerated serum clearance compared to full-sized antibodies, making it particularly favorable for in vivo imaging. IAB22M2C conjugated to the chelator desferrioxamine (Df) and radiolabeled with Zirconium-89 (^89^Zr) has been successfully used in a number of preclinical and clinical trials with a focus on cancer immunotherapy applications (*26*, *27*). With a long radioactive half-life of 78.4 hours, ^89^Zr allows the tracer’s biodistribution to be followed for several days post-injection (p.i.). However, because of its long half-life, radiation dose concerns have prevented wider application of ^89^Zr-immunoPET in non–life-threatening disease and healthy populations. The advent of total-body PET scanners covering all or much of the body (*28*), which offer a radiation detection sensitivity increase of one to two orders of magnitude compared to conventional PET scanners (*29*), enables high signal-to-noise ratio imaging of ^89^Zr-based radiotracers at substantially lower injected doses in addition to capturing kinetics across all the organs and tissues of interest. This enables complete characterization of the pharmacokinetics of these immunological PET tracers across a wide range of applications (*28*, *30*). Total-body PET is currently the only available technology that allows noninvasive in vivo measurements of T cell distribution and kinetics inside all tissues in human subjects, with acceptable radiation dose burden. In this work, low (<20 MBq) doses of ^89^Zr-Df-Crefmirlimab tracer were used with the 194-cm-long uEXPLORER total-body PET scanner to study the biodistribution and kinetics of CD8^+^ cells in COVID-19 convalescent patients and in healthy controls.

## RESULTS

Eight subjects were enrolled, including five COVID-19 convalescent patients and three healthy controls. The demographics of the study participants are included in Table 1. All subjects had received at least one dose of a COVID-19 mRNA vaccine before their first imaging session, except for one healthy individual (Sub08) who had not received any vaccination prior or during the imaging study. The participants varied in vaccination timeline with respect to their imaging sessions, and one patient (Sub01) contracted COVID-19 before vaccination. Patients with COVID-19 were possibly exposed to different variants of the virus with infection timelines ranging from January 2021 to March 2022 and varied in terms of infection symptoms and past medical history (table S1). The last two enrolled patients (Sub04 and Sub05) had the mildest symptoms, with exposure timelines during or after the Omicron variant surge in California.

**Table 1.** Demographics of study participants. BMI, body mass index.

Demographic	COVID-19	Control
Total participants (*n*)	5	3
Age range (y)	27–51	25–59
Sex (*n*)		
Male	0	2
Female	5	1
BMI range (kg/m^2^)	20–43	21–31

The mean injected radiotracer activity was 18.8 MBq (0.51 mCi), with a range of 15.4 to 21.8 MBq (0.42 to 0.59 mCi). The mean injected minibody mass was 1.50 mg, with a range of 1.33 to 1.77 mg. The injections and PET imaging were well tolerated, with no adverse reactions to the infusion. No adverse effects and no clinically notable changes in vital signs were observed during the study. Two COVID-19 convalescent patients did not have dynamic scans, and the dynamic scan of one control subject was terminated early at 65 min due to patient motion and discomfort.

### Blood clearance of the radiotracer

The whole blood clearance was best described by a triexponential model, with an initial half-life of 5.1 ± 2.2 min (range, 2.6 to 8.1 min), an intermediate half-life of 55.9 ± 28.0 min (range, 29.3 to 120.0 min), and a terminal elimination half-life of 22.1 ± 11.8 hours (range, 13.4 to 50.4 hours). One COVID-19 convalescent patient (Sub02) showed significantly longer terminal half-life compared to all other subjects (fig. S1).

### Total-body biodistribution of the radiotracer

**Fig. 1.**
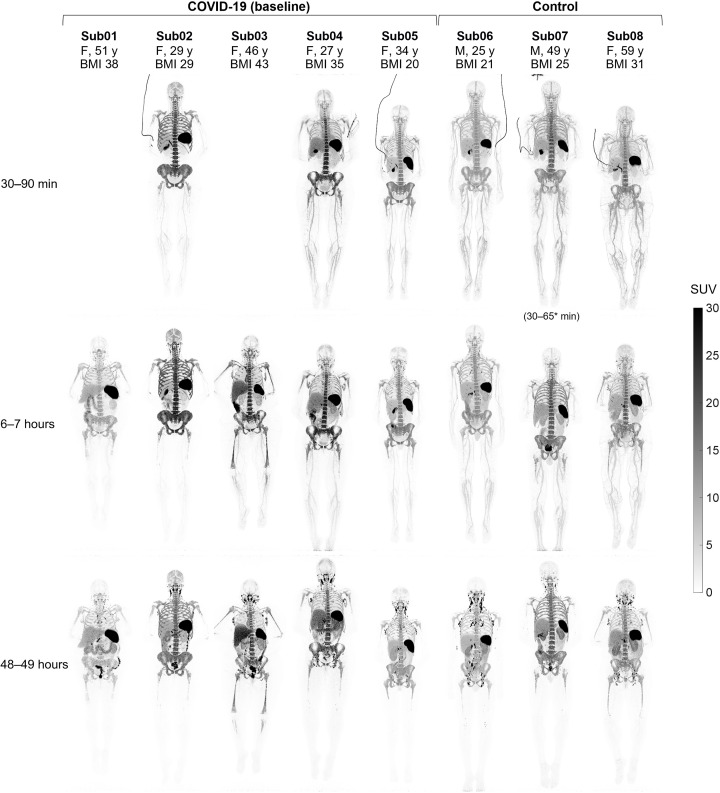
Maximum intensity projection (MIP) of decay-corrected SUV images of the baseline scans. The baseline scans of COVID-19 convalescent patients and healthy control subjects are compared at three imaging time points. Sub01 and Sub03 skipped dynamic imaging. M, male; F, female, y, years; BMI, body mass index.

**Fig. 2.**
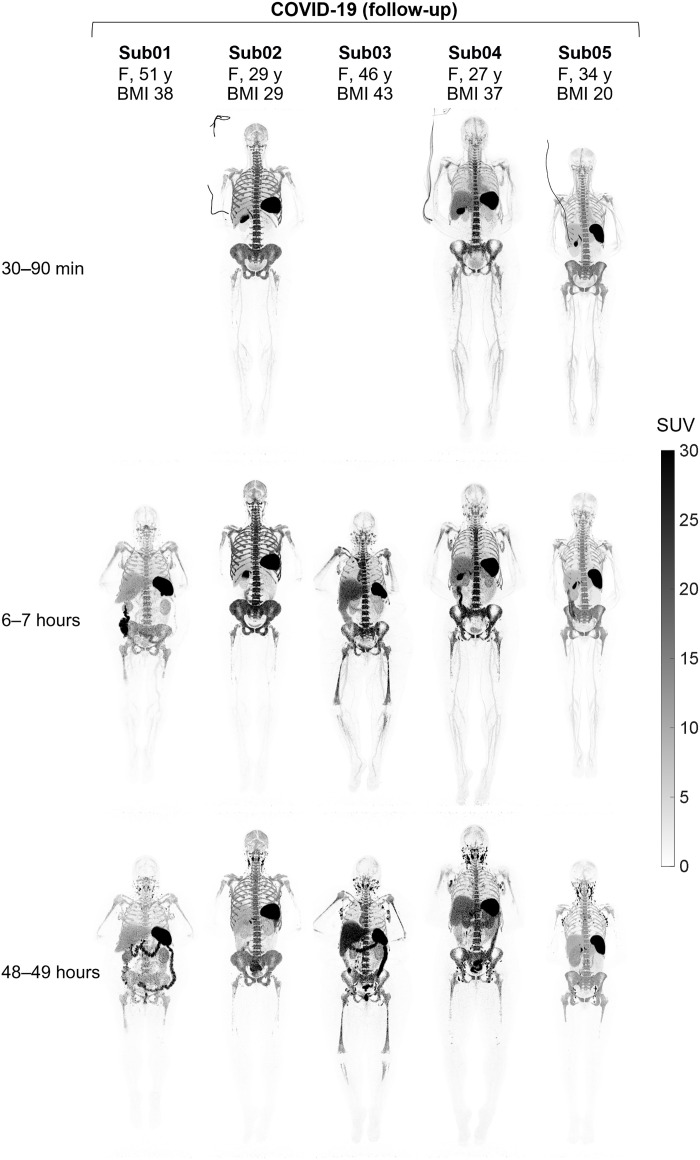
MIP of decay-corrected SUV images of the 4-month follow-up scans. The follow-up scans of the COVID-19 convalescent patients are shown at three imaging time points. Sub01 and Sub03 skipped dynamic imaging.

Standardized uptake value (SUV) images of the baseline scans of COVID-19 convalescent patients and healthy controls at three imaging time points showed high uptake in lymphoid organs of all subjects (Fig. 1), with the highest uptake observed in the spleen of all subjects, followed by the bone marrow, liver, tonsils, and lymph nodes. Bone marrow uptake was particularly prominent in the vertebrae, sacrum, ilium, ribs, sternum, clavicle, and scapulae of all subjects and showed variable extended lengths in humeral and femoral shafts. Peripheral lymph nodes showed marked uptake in all subjects as early as 30 to 90 min p.i. and peaked at the 48-hour time point. Prominent uptake was observed in head and neck lymph nodes of all subjects, and a subset of subjects also showed uptake in their axillary, pelvic, mediastinal, as well as upper and lower limb lymph nodes. Consistent with the expected hepatobiliary clearance of the radiotracer, the gallbladder was visualized during the dynamic scans of all subjects, except for the two COVID-19 convalescent patients who did not have dynamic scans, one of which (Sub03) had a history of cholecystectomy. Excluding Sub03, the gallbladder was still visualized in 9 of 11 scans performed at each of the 6- and 48-hour time points. Furthermore, all participants showed activity in their bowel during the 48-hour time course of the study, with the largest activity observed in the colon and rectum. Cross-sectional analysis suggested that the activity was in the large bowel lumen, supported by changes in the location of the activity over the 48 hours. The small bowel also contained low levels of activity; however, it was not possible to confirm whether the activity was in the lumen or in the Peyer’s patches of the small bowel wall. Muscle, cerebrum, and cerebellum uptakes were low in all subjects, with SUV_mean_ values below 0.2, 0.7, and 0.8, respectively, at all time points. Lymphoid tissues in the nasal and pharyngeal area had visible uptake in all subjects with similar range of values. Comparing the baseline images of the COVID-19 convalescent patients to the images from their 4-month follow-up scans (Fig. 2) showed that despite the observed differences and heterogeneity among the study participants, irrespective of their study group, the follow-up scans of each patient exhibit notable similarities to their baseline scans particularly in the bone marrow.

### Kinetics during the 48 hours of imaging

**Fig. 3.**
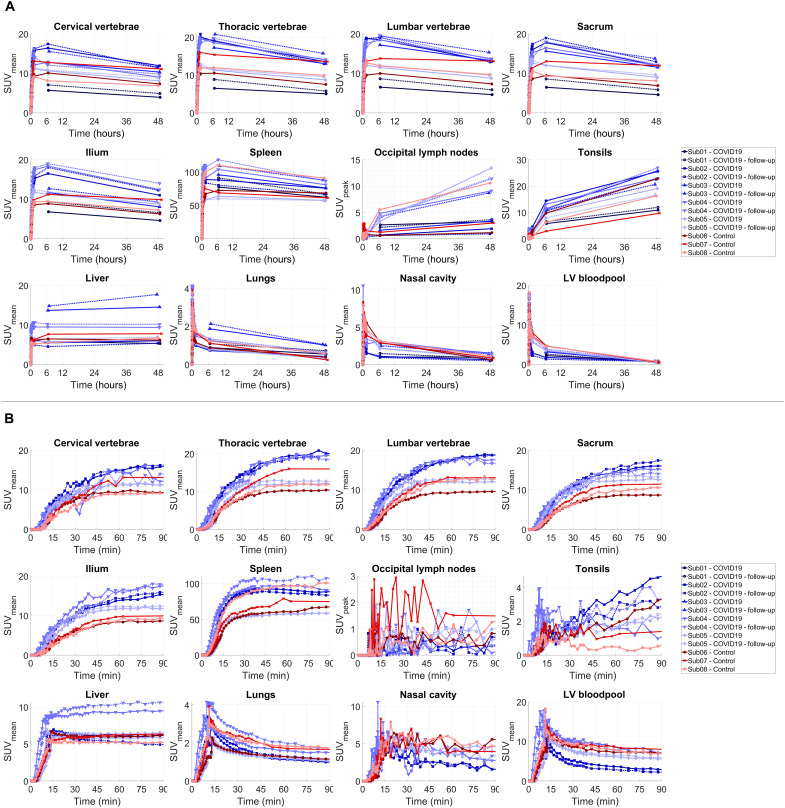
Decay-corrected TACs of different organs of interest. (**A**) TACs representing the delivery, retention, and clearance of the tracer over the 48-hour time course of the study are shown for the bone marrow (cervical, thoracic, and lumbar vertebrae; sacrum; and ilium), spleen, liver, lymph nodes, tonsils, lungs, nasal cavity, and the left ventricle (LV) blood pool, in addition to (**B**) zoomed-in plots on the first 90 min after tracer administration for all subjects. Control and COVID-19 subjects are in shades of red and blue, respectively. The lymph node TACs show an example lymph node selected from the occipital region of each subject [PET/computed tomography (CT) images shown in fig. S2]. The occipital region was selected as a common area where all subjects showed quantifiable uptake, and the TACs were not affected by spillover from adjacent lymph nodes or blood vessels.

The time activity curves (TACs) from all investigated organs of interest showed consistent trends in tracer kinetics in all subjects (Fig. 3). Different regions of the bone marrow showed an increasing trend during the 90-min dynamic scans in all subjects, with a plateauing rate of uptake toward 90 min. Spleen TACs also showed a plateauing increasing trend during the 90-min dynamic scans, except in two COVID-19 subjects (Sub02 and Sub04), in which the TACs showed a decrease after peaking at around 1 hour. Lymph nodes visualized with high contrast at 6- and 48-hour time points could mostly be visualized also on the 30- to 90-min images with SUVpeak values in the range of 0.2 to 5.2. Between the 6- and 48-hour time points, all subjects showed a decrease of SUV in the spleen, bone marrow, and lungs and parallel increase of SUV in the lymph nodes and tonsils, which was quantified by the percentage change in SUV at the 48-hour time point relative to the 6-hour time point in all investigated organs of interest (fig. S3). Comparing the percentage change in SUV during the last 42 hours showed similar trends in all organs of interest in all subjects, with no significant difference between the COVID-19 and the control group. Liver uptake showed relatively smaller changes during the last 42 hours, with an increasing trend in three subjects.

**Fig. 4.**
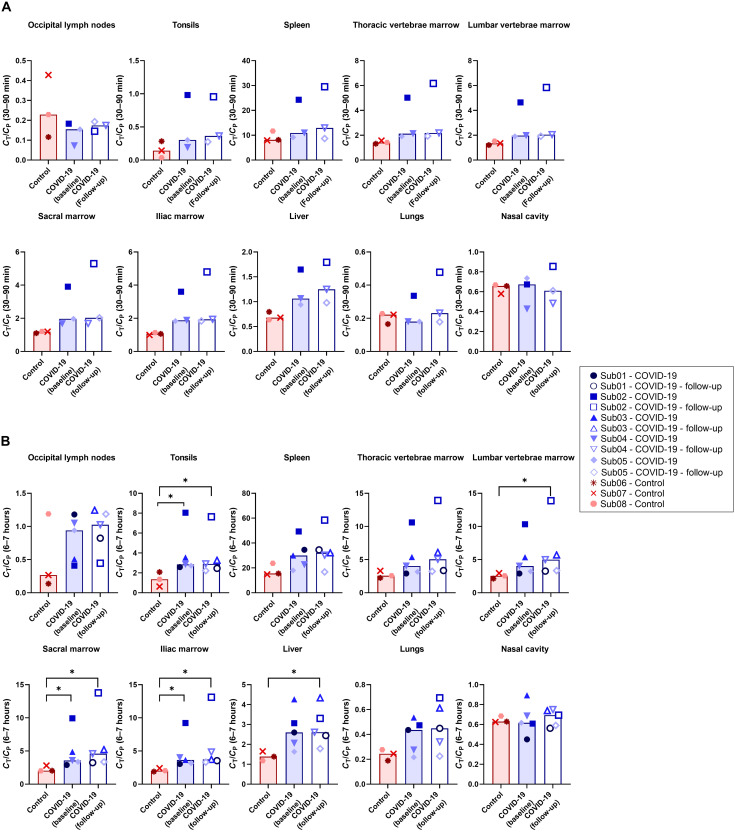
TBRs of different organs of interest. TBRs are compared between COVID-19 and control subjects in the lymph nodes, tonsils, spleen, bone marrow, liver, lungs, and nasal cavity (**A**) during the 30- to 90-min dynamic scans and (**B**) at the 6- to 7-hour time point. Bars represent the median in each group (*P = 0.036).

Tissue-to-blood ratio (TBR) curves plotted as a function of time for different regions of the bone marrow and liver showed a separation between the COVID-19 and the control group during the 90-min dynamic scans up to the 7-hour time point, with higher values observed in the COVID-19 group (fig. S4). The sacrum and ilium bone marrow showed the largest differences between the two groups. Comparing the TBRs of all subjects at 30 to 90 min and 6 to 7-h time points (Fig. 4) showed significant differences between the COVID-19 and the control group at the 6 to 7-hour time point in the liver, different bone marrow regions, and tonsils (*P* = 0.036). Moreover, TBRs of all bone marrow regions, spleen, and tonsils were two to three times higher in one COVID-19 subject (Sub02) than all other patients during the first 7 hours. No significant difference was observed in the spleen, lungs, or nasal cavity between the two groups.

**Fig. 5.**
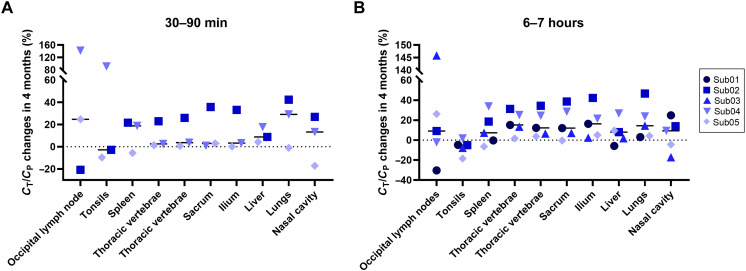
Longitudinal changes of TBRs in different organs of interest of COVID-19 convalescent patients. Percentage changes of TBR at 4-month follow-up scans of the COVID-19 convalescent patients relative to their baseline scans is shown in the lymph nodes, tonsils, spleen, bone marrow, liver, lungs, and nasal cavity at (**A**) 30 to 90 min and (**B**) 6 to 7 hours.

Comparing the percentage changes of TBR at 4-month follow-up scans relative to the baseline scans of the COVID-19 convalescent patients (Fig. 5) did not show consistent trends in most organs of interest, except for the bone marrow, in which a consistent trend toward increased TBR was observed in all bone marrow regions of the COVID-19 subjects during the first 7 hours of imaging. While this increasing trend in bone marrow TBRs ranged from 2 to 42% at the 6-hour time point in four of five COVID-19 subjects, in one COVID-19 subject (Sub05), the follow-up scans did not show substantial changes in the bone marrow regions, with only 0 to 5% changes compared to the baseline scan.

### Kinetic modeling approaches for ^89^Zr-Df-Crefmirlimab

Conventional PET kinetic modeling approaches based on one-tissue (1T) and two-tissue (2T) compartmental models with Akaike information criterion (AIC) model selection successfully fitted the TACs in the lungs, spleen, bone marrow, tonsils, and selected occipital lymph nodes (fig. S5 and table S2). In all cases, all available time points were used for model fitting, and using the earlier time points alone was not sufficient to accurately model the TACs at later time points. Visual inspection of the AIC-preferred model fits in all organs of interest suggested low residual errors in fitting all imaging time points (figs. S6 and S7), except for the lymph nodes, in which TACs were more affected by statistical noise due to small number of voxels included in the analysis. Normalized sensitivity plots (fig. S8) showed increasing sensitivity of all model microparameters up to the 48-hour time point, except for *v*_b_ and *K*_1_, which reached their maximum sensitivity at earlier time points in some cases. Correlations observed between microparameters of the 2T model (table S3) were mostly expected and similar to those commonly observed in conventional PET kinetic modeling. Simulations of the TAC noise model in all organs of interest showed low biases in all microparameter estimations (table S4), suggesting small effects from statistical noise and high confidence in microparameter estimation at organ-level, with the exception of lungs that showed increased errors compared to other organs. In all investigated organs, the slope of the Patlak plot changed from 90 min to 48 hours and because only two data points were available at later time points, it was not possible to determine the equilibrium time (fig. S9).

In lungs, AIC favored the 2T model over 1T3P model in all subjects; however, the AIC values were close between 2T4P and 2T5P models in many cases (fig. S5). All three models could fit the early 90-min data points, but the fitting errors were larger for the late time point data with the 1T3P model (figs. S6 and S7). Air fraction correction was not applied to the results, as using the low-dose computed tomography (CT) images resulted in overestimation of air fraction values, particularly in high–body mass index (BMI) subjects. With no air fraction correction, no significant difference was observed between the control and COVID-19 groups and only one COVID-19 convalescent patient (Sub02) showed increased *K*_i_ values.

In the spleen, AIC largely favored the 2T5P model in all subjects (fig. S5). 2T4P and 1T3P models could not fit the later time points, and increasing the weighting factors of the late time points resulted in inaccurate fits on the first 90-min data (fig. S10). *K*_1_ and *k*_2_ were highly correlated (>98%) with *v*_b_, and increase in *K*_1_ up to its upper bound was compensated by a decrease in *v*_b_. Therefore, *v*_b_ was set to 0.4 during the fitting for spleen in all cases. The fitted model suggested that the concentration of the bound tracer in the second compartment increases up to 12 to 24 hours p.i. and starts to drop thereafter (fig. S11). No significant difference was observed between the two groups.

**Fig. 6.**
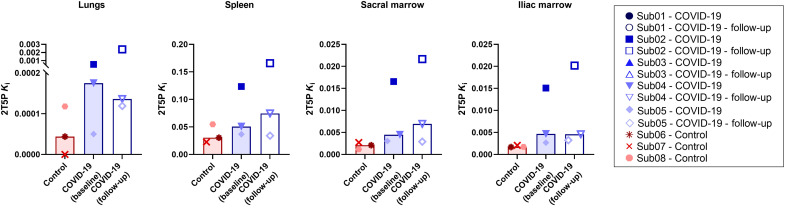
Net influx rate of the 2T5P model. Net influx rate (*K*_i_) obtained by 2T5P model fits in the lungs, spleen, and sacrum and ilium bone marrow are compared in all subjects with dynamic scans. Bars represent the median in each group.

In the bone marrow, AIC favored the 2T5P model in all subjects (fig. S5). The 2T4P model could fit the data from all time points but with higher AIC values, whereas the 1T3P model could not fit the 48-hour time point. The microparameter estimates for the 2T5P model were similar in the sacrum and ilium. 2T5P model *K*_i_ values were higher in the bone marrow of COVID-19 convalescent patients compared to the controls (*P* = 0.1) (Fig. 6). Similar to the spleen, the fitted 2T5P model suggested increasing concentration of the bound tracer up to 12 to 24 hours p.i. and a decrease thereafter.

In the tonsils, AIC favored the 1T3P model, and in the lymph nodes, AIC favored the 2T4P and 1T3P models interchangeably (fig. S5). In many cases, the 2T model microparameters were zero or resulted in inconsistent estimates; therefore, 1T3P model was selected for the lymph nodes as well.

### Uptake in the thymus

**Fig. 7.**
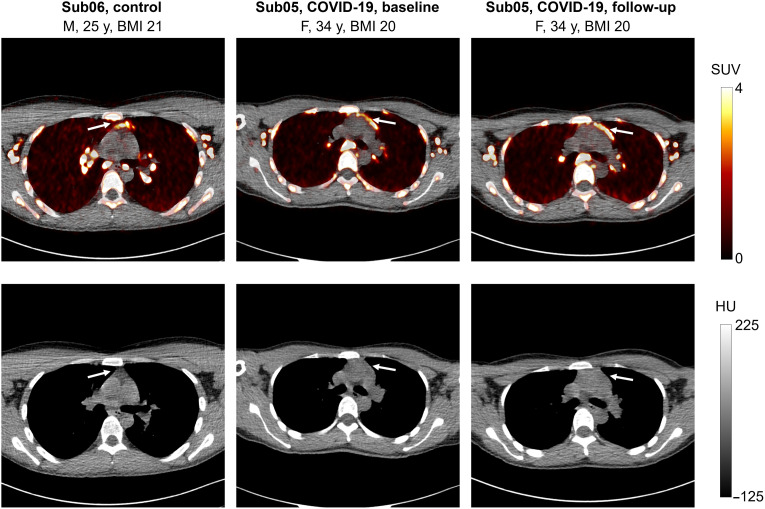
PET/CT image slices of thymus uptake. Selected transverse PET/CT image slices of one control subject and one COVID-19 convalescent patient (at baseline and follow-up scan) showing thymus uptake at 48-hour time point of imaging. HU, Hounsfield unit.

Thymus uptake was observed only at the 48-hour time point in two subjects (Fig. 7), including one COVID-19 convalescent patient (in both baseline and follow-up scans) and one control subject, with SUV_mean_ values of 4.0 and 4.7, respectively. The COVID-19 and control subjects were 34 and 25 years old, respectively, and had the lowest BMIs among all subjects, with BMIs of 20 and 21 kg/m^2^, respectively. The thymus fatty degeneration scoring based on low-dose CT images in all subjects (fig. S12) showed solid thymic gland with a score of 3 in the COVID-19 convalescent patient and half fatty and half soft tissue attenuation with a score of 2 in the control subjects. Two other under–30-year-old subjects in the study (Sub02 and Sub04) also showed solid thymic glands with a score of 3. Both subjects were in the COVID-19 group and had higher BMIs compared to the two subjects that showed thymus uptake in their PET images.

### Peripheral blood assays

**Fig. 8.**
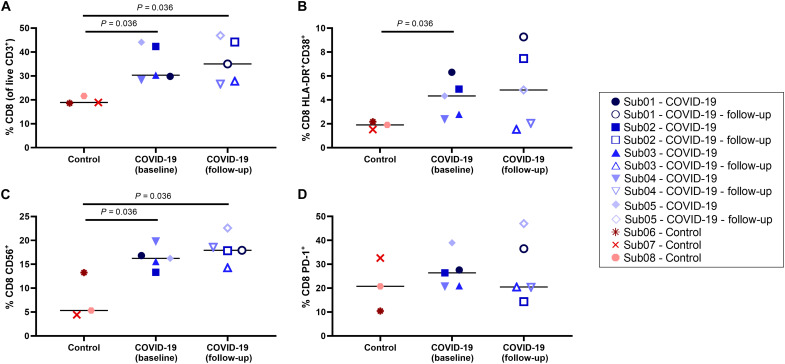
Peripheral blood CD8^+^ T cell phenotyping. (**A**) Percentage of CD8^+^ T cells within the live CD3^+^ population, (**B**) percentage of activated CD8^+^ T cells characterized by CD38 and HLA-DR coexpression and (**C**) CD56 expression, and (**D**) percentage of exhausted CD8^+^ T cells characterized by PD-1 expression are compared in all subjects.

CD8^+^ and CD4^+^ T cell immunophenotyping results (Fig. 8 and fig. S13, respectively) showed an increase in percentage of CD8^+^ T cells and a decrease in percentage of CD4^+^ T cells in COVID-19 convalescent patients compared to the controls (*P* = 0.036). Moreover, an increased frequency of activated CD8^+^ T cells was observed in COVID-19 convalescent patients, both in CD38^+^ human leukocyte antigen-DR (HLA-DR)^+^ cells and CD56^+^ cells (*P* = 0.036). No difference was observed in frequency of programmed cell death protein 1 (PD-1)^+^ cells between the two groups.

Comparing the percentage of memory subsets of CD8^+^ and CD4^+^ T cells in all subjects (fig. S14) showed a trend toward higher frequency of CD8^+^ effector memory T cells in the COVID-19 convalescent patients compared to the controls. The percentage of mucosa-associated invariant T (MAIT) cells, regulatory T (T_reg_) cells, natural killer (NK) cells, and B cells compared between the two groups (fig. S15) showed a trend toward higher percentage of MAIT cells in COVID-19 convalescent patients (*P* = 0.072), and no significant difference was observed between the two groups with other cell types.

The total percentage of CD8^+^ and CD4^+^ memory T cells responding in any way [CD107a, interferon-γ (IFN-γ), interleukin-2 (IL-2), macrophage inflammatory protein–1β (MIP-1β), or tumor necrosis factor–α (TNF-α)] to SARS-CoV-2 spike and nucleocapsid proteins compared in all subjects (fig. S16) showed trends toward higher-magnitude CD8^+^ and CD4^+^ responses to both spike and nucleocapsid in COVID-19 convalescent participants compared to the controls, which was significant for the spike-specific CD4^+^ responses (*P* = 0.036), but no significant differences were observed between the baseline and 4-month follow-up scans. Furthermore, CD4^+^ responses were of higher magnitude than CD8^+^ responses. Individual responses in CD8^+^ and CD4^+^ memory T cells (figs. S17 and S18, respectively) showed potential trends toward increasing spike-specific CD8^+^ T cell degranulation response (CD107a^+^), higher frequencies of spike-specific CD4^+^ IL-2^+^ T cells, and higher frequencies of spike-specific CD4^+^ IFN-γ^+^ T cells in COVID-19 convalescent patients (*P* = 0.036). SARS-CoV-2–specific CD8^+^ and CD4^+^ responses were mainly dual and monofunctional (figs. S19 and S20, respectively), and CD4^+^ T cell responses were slightly more polyfunctional than CD8^+^ responses. Only a slight increase in polyfunctionality could be observed for nucleocapsid-specific CD8^+^ T cells in COVID-19 convalescent participants at the 4-month time point.

Last, Spearman correlation analysis showed moderate correlations (*r* in the range of 0.57 to 0.68) between peripheral blood CD8^+^ immunophenotyping results and TBRs from the 6-hour time point in the spleen (*r* = 0.59 with CD8^+^ HLA-DR^+^CD38^+^, and *r* = 0.67 with total CD8^+^ memory), thoracic vertebrae marrow (*r* = 0.61 with CD8^+^ TEMRA), lumbar vertebrae marrow (*r* = 0.57 with CD8^+^ TEMRA), lungs (*r* = 0.60 with total CD8^+^ memory), and nasal cavity (*r* = −0.68 with CD8^+^ PD-1^+^ and *r* = 0.58 with CD8^+^ T_EM_) with *P* values below 0.05 (fig. S21). A strong correlation was observed between the nasal cavity TBRs and CD8^+^ TEMRA cells in the peripheral blood (*r* = 0.71, *P* = 0.01). It has to be noted that none of the correlations were found to be significant after including a Bonferroni multiple comparison correction to the 80 correlation tests performed.

## DISCUSSION

We present the first results on the biodistribution and kinetics of a CD8-targeted radiotracer in healthy control individuals and in subjects following a viral infection. The biodistribution and general trends were in good agreement with previous oncologic human studies using ^89^Zr-Df-Crefmirlimab (*26*, *27*). Although there was a sixfold reduction in injected radiation dose compared to previous phase I human studies, significant image quality improvements, both in terms of noise reduction and higher contrast in small structures, were observed in the total-body PET scans compared to the previous human PET scans (*26*, *27*). Notably, a large number of high-contrast lymph nodes were visualized in all subjects and the tracer kinetics in any given organ followed consistent trends in all subjects. Considering the low positron fraction of ^89^Zr, the remarkable image quality obtained in this study demonstrated feasibility of high-quality dynamic imaging with ^89^Zr-labeled immunological tracers across the whole body, at doses that permit longitudinal imaging in healthy individuals and any disease state.

This study revealed significant differences in the bone marrow CD8 concentrations of COVID-19 convalescent patients compared to controls, with increased TBRs in the first 7 hours of the study and increased net influx rates (2T5P *K*_i_) observed in the bone marrow of COVID-19 convalescent patients. These changes were not evident in the commonly used SUV images because the measured SUV cannot account for the time-varying tracer concentration in the blood and tissue compartments, nor the effects of cell trafficking. This study demonstrates the role of dynamic imaging and kinetic modeling in providing quantitative biomarkers. While SUV TACs from the first 90 min showed higher values in the sacrum and ilium bone marrow of the COVID-19 convalescent patients compared to controls, SUV TACs overlapped between the two groups for other bone marrow regions, particularly at later time points where the two additional COVID-19 subjects with no dynamic scans were included. Plotting TBRs as a function of time, however, showed more distinct differences between the two groups in all bone marrow regions up to the 7-hour time point. The TBR curves from the three control subjects showed very similar values, particularly in the sacrum and ilium, while within the COVID-19 group, one subject (Sub02) who got infected with COVID-19 twice showed consistently significant higher values in all bone marrow regions compared to all subjects. Other COVID-19 convalescent patients showed a separate cluster of results but still higher than the controls. To ensure that the statistical comparisons between the two groups were not heavily driven by one participant (Sub02), the statistical tests were repeated in two settings: (i) reducing the sample size and removing Sub02 from the COVID-19 group and (ii) keeping the sample size constant and replacing the data of Sub02 with the minimum values within the COVID-19 group. In the first setting, *P* values with initial values of 0.036 increased to 0.057, and in the second setting, *P* values with initial values of 0.036 remained unchanged. Furthermore, it has to be noted that despite the promising results of kinetic modeling, the statistical significance of the 2T5P *K*_i_ comparisons were affected by the smaller sample size compared to late time point TBR data due to unavailability of dynamic data for two COVID-19 convalescent patients.

Tumor-to-blood ratio has been previously shown to be a surrogate for the net metabolic rate of other radiotracers such as ^18^F-fluorodeoxyglucose in tumors (*31*). Comparing TBRs among different subjects at a specific time point, while equilibrium has not been reached, requires extra care, as TBR changes as a function of time and is affected by blood clearance rates. Plotting TBR as a function of normalized time in Patlak plots accounts for variations in blood clearance. The similarity of the TBR plots (Fig. 3) to Patlak plots (fig. S9) during the first 7 hours is consistent with similar initial and intermediate blood clearance rates (fig. S1) among all subjects. Patlak plots also reflect the differences in terminal blood clearances and show improved separation between the two groups at later time points. Furthermore, TBRs from the 6-hour time point were highly correlated with net influx rates (*K*_i_) obtained from 2T5P fits on the complete 48-hour TACs (ρ > 0.99 in the bone marrow and spleen and ρ > 0.93 in the lungs), suggesting that they can be used as surrogates for *K*_i_. The mitigated separation between the two groups at later time points in the Patlak plots may be attributed to increased cell trafficking effects that are not accounted for. Future studies should investigate correlation of early-time point TBRs with model macroparameters, best describing the CD8 tissue density in the absence of cell trafficking effects.

In addition to higher CD8 TBRs values observed in the bone marrow of COVID-19 convalescent patients compared to controls, flow cytometry results also showed higher percentage of CD8^+^ T cells, higher percentage of activated CD8^+^ T cells, and higher percentage of CD8^+^ memory T cells in the peripheral blood of COVID-19 convalescent patients compared to controls. Furthermore, the two longitudinal scans performed on COVID-19 convalescent patients showed consistently increasing trends in the bone marrow of all subjects between 2 and 6 months after infection. While the magnitude of these longitudinal changes varied among different patients, low degree of heterogeneity was observed in different bone marrow regions of each patient. These results are consistent with previous reports on selective accumulation of virus-specific CD8^+^ T cells in the bone marrow of mice and humans (*32*–*37*). Murine studies have reported an increase in the frequency of memory CD8^+^ T cells in the bone marrow after recovery from viral infection (*32*), and it has been shown that the pool of CD8^+^ memory T cells in the bone marrow is not size-restricted and can readily expand when the host is exposed to new antigenic challenges (*33*). Bone marrow has been identified as a major pool and the preferred site for proliferation of memory CD8^+^ T cells following a viral infection (*34*). Furthermore, a recent study on patients infected with the mumps virus (MuV) reported significantly increased expression of CXCR4 (derived as a bone marrow–homing marker) in MuV-specific CD8^+^ T cells of the peripheral blood between 1.5 and 9 months after infection (*35*).

The peripheral blood flow cytometry data, on the other hand, only showed a nonsignificant population-based increase in the median of the 4-month follow-up scans compared to the baseline scans. Although a high degree of heterogeneity can be expected among human subjects, the large variations observed within each group may also be partly due to the limited precision of the flow cytometry methods, which usually require larger cohort of subjects for making statistically meaningful conclusions. These limitations suggest that flow cytometry methods may be inadequate for studying small longitudinal changes in individual subjects. These large variations can be also observed in the reported results from previous studies using the peripheral blood of COVID-19 convalescent patients, in which a general trend toward declining SARS-CoV-2–specific CD8^+^ memory T cell response is observed from statistical analysis on the peripheral blood of two large cohort of patients between 1 and 8 months after infection, but inconsistent findings are observed in longitudinal measurements in individual subjects (*9*, *10*). The small number of participants in this pilot study makes it difficult to conclude whether the observed trends in increased percentages of CD8^+^ T cells in the peripheral blood of COVID-19 convalescent patients at the 4-month follow up scans are due to the heterogeneity of patients with COVID-19, clinical differences among the subjects of this study with those of previous studies, or methodological variations. Furthermore, while vaccination timeline is expected to affect the spike-specific responses, some inconsistencies can be observed in SARS-CoV-2–specific responses, such as the high spike-specific and nucleocapsid-specific responses in the unvaccinated control subject (Sub08), which could be due to cross-reactivity with seasonal coronaviruses or possible subclinical infection or exposure. This raises important questions on feasibility and challenges of recruiting negative controls in modern COVID-19 studies. Nevertheless, image-based results show no significant difference in the bone marrow of the unvaccinated subject compared to other control subjects, but higher TBRs, in similar range to COVID-19 subjects, can be observed in the spleen of the unvaccinated subject, which needs further investigation.

Kinetic modeling based on conventional PET compartmental models showed reproducible results at organ level, which were in good agreement with immunobiology of the investigated organs, and the expected T cell–trafficking effects were reflected in the kinetic model selection results. Although the conventionally used one-tissue and two-tissue compartmental models do not include separate pathways for the trafficking of radiolabeled cells, and therefore, they are not expected to accurately represent the kinetics of the ^89^Zr-Df-Crefmirlimab in lymphoid tissue, in the absence of cell trafficking, the main mechanism of uptake can be simplified and approximated by the 2T4P model, in which upon entrance of the free ^89^Zr-labeled minibody tracer into tissue, the tracer binds to the CD8 receptors and gets irreversibly internalized within the cell. In the presence of cell trafficking, which is non-negligible over the 0 to 48-hour time frame in lymphoid organs, all rate constants of tracer exchange between the model compartments will include components from trafficking and the model will require a non-zero *k*_4_ to represent the trafficking of radiolabeled CD8^+^ T cells out of the tissue. This is reflected in the AIC model selection results for the spleen, bone marrow, and lungs. The 2T5P model, which includes a non-zero *k*_4_, is largely favored in the spleen, where a large population of naïve recirculating CD8^+^ T cells are expected and the migration of radiolabeled CD8^+^ T cells out of spleen may prominently contribute to the *k*_4_. A similar effect can be observed in the bone marrow, but to a lesser extent. In the case of healthy lung tissue, relatively low concentrations of CD8^+^ T cells are expected and a large fraction of the CD8^+^ T cell population could be noncirculating tissue-resident T cells. As a result, the difference between the 2T5P and 2T4P models becomes much smaller, particularly in the healthy individuals where 2T4P is favored, which could be attributed to a smaller effect from T cell trafficking in these cases.

Furthermore, the effects of blood flow and permeability of the blood vessels to the 80-kDa minibody molecules in different types of tissue could explain the differences observed in the TACs of different organs and their corresponding tissue compartments (fig. S11). The presence of sinusoidal capillaries in the spleen and bone marrow, which allow exchange of large molecules between blood and the surrounding tissue, in addition to the high blood flow in these two CD8-rich organs results in fast entrance of the free tracer into the tissue, followed by a slower process of binding within the tissue in the second tissue compartment. While a continuous exchange of labeled CD8^+^ cells between tissue and blood is present in parallel, the high permeability and blood flow could result in saturation of binding sites in these two organs as reported in a previous dose escalation study when a 1.5-mg minibody mass dose is used (*27*). The following decrease in the concentration of the second tissue compartment (fig. S11) can therefore be attributed to the flux of radiolabeled CD8^+^ T cells out of the tissue. In the case of lungs, lower permeability, lower blood flow, and lower concentrations of CD8^+^ T cells can be expected than the spleen or bone marrow, and as a result, a large fraction of the initial signal in the lungs is due to tissue blood fraction and low levels of free tracer entering the tissue, followed by slow binding and clearance. In the case of lymph nodes and tonsils, where high concentrations of binding sites are expected but tracer delivery to the tissue may be slower due to significantly lower blood flow compared to the spleen, bone marrow, and lungs, AIC favors the 1T3P model in which the low amounts of free tracer slowly entering the tissue bind relatively quickly to the highly available binding sites in the tissue. However, the increase of uptake in lymph nodes and tonsils over time may also be largely attributed to migration of radiolabeled T cells to these tissues, which cannot be separated from the free tracer uptake in the current model. Note that the whole blood compartment itself contains CD8^+^ cells, and as these cells get radiolabeled with the tracer and are in continuous exchange with the radiolabeled CD8^+^ cells in different lymphoid organs, the concentration of radiolabeled CD8^+^ cells in whole blood changes as a function of time. Future studies should investigate these changes in the whole blood compartment as a function of time and incorporate them either directly in the compartmental model or indirectly with correction factors to separate the free tracer uptake kinetics from cell-trafficking kinetics. In the case of lymph nodes in particular, a second distinct uptake pathway may also be present through afferent lymphatic vessels, which needs further detailed investigation. Previous studies on the anatomy of the lymphoid compartment within lymph nodes have described a 70-kDa size exclusion limit restricting access of heavy weight molecules to lymph node T cell zones through lymph (*38*). However, there has been ongoing research on identifying special routes of transport for high–molecular weight molecules into the conduit system of the lymph nodes (*39*).

Normalized sensitivity plots suggest that 48 hours of imaging is required for estimating the model microparameters and might be sufficient. However, more intermediate imaging time points are required to study this in detail. The Patlak plots further illustrate this need, where even in the case of the healthy lung tissue in which AIC favors the 2T4P model, the equilibrium time seems to be larger than 7 hours and cannot be determined from the current dataset. Furthermore, although simulations of the TAC noise model show very promising results, particularly in the spleen and bone marrow with low bias values in the range of ±1% for most microparameters, it is still possible that the noise model was underestimated for the later time points, because only two data points were available for fitting and the model fits might have been affected by overfitting. This can also be investigated in future studies with more intermediate imaging time points.

While the kinetics of the radiotracer are substantially different in the liver compared to the bone marrow and, conventionally, liver is not considered a lymphoid organ, TBR curves in the liver also show higher values in the COVID-19 subjects compared to the controls and substantially higher values for Sub02. This could be due to the presence of subsets of CD8^+^ T cells in the liver, the population of which may be affected during viral infection. However, because of the hepatobiliary clearance of the radiotracer and dual blood supply of the liver, further studies are required to accurately model the kinetics of the tracer in the liver and quantify the cell-trafficking effects. Furthermore, TBR curves in the lungs show overlapping curves between the two groups, which may be expected since as all COVID-19 convalescent patients in the study had relatively mild infection, with no hospitalization and no findings on lung involvement. However, no certain conclusions can be made as air fraction correction was not applied to the data due to low-quality of CT acquisitions. The systematic significant differences observed in all tissue concentrations of one COVID-19 convalescent patient (Sub02), which is not evident in the peripheral blood results, need further investigation with respect to their clinical record and history of two COVID-19 infections, including an asymptomatic infection after one vaccination dose and a second infection after 3 months with significant illness and respiratory symptoms.

The thymus uptake observed in the two lowest-BMI subjects, both under 30 years old, is consistent with previous reports on increased fatty degeneration in the thymus with aging and high BMI (*40*). While fatty degeneration scoring based on CT images shows predominant soft tissue attenuation in two higher BMI subjects under 30 years old, with score 3, no thymus uptake is observed in their corresponding PET images. Furthermore, observing the thymus uptake only at the 48-hour time point not only suggests very low permeability or blood flow but also could be related to previous preclinical findings suggestive of a blood-thymus barrier restricting access of high–molecular weight particles from the blood to T cells in the thymus (*41*, *42*). In addition, while most CD4^+^ and CD8^+^ T cells in the thymus are recently generated naïve T cells, preclinical studies in rodents, lamb, and pigs suggest that a small fraction of thymic mature T cells are immigrants from periphery (*43*), and therefore, migration of these mature CD8^+^ T cells, previously labeled with the radiotracer, back to the thymus could further explain the thymus uptake only at the 48-hour time point.

This pilot study had some limitations, particularly with a small number of participants. Therefore, the differences observed between COVID-19 convalescent patients and healthy controls, as well as the longitudinal changes in COVID-19 convalescent patients, need to be further investigated with larger sample sizes. CD8-targeted imaging with ^89^Zr-Df-Crefmirlimab is in general limited to targeting all CD8^+^ cell types—with no specificity to CD8^+^ T cells or their subsets—and can include effects from changes both in CD8 expression and CD8^+^ cell population. Furthermore, it cannot distinguish between the relatively small percentage of antigen-specific CD8^+^ T cells, which are directly affected by the viral infection and its recovery process, and the vast majority of CD8^+^ T cells that do not recognize the antigen but which may have been nonspecifically activated due to the host inflammatory response through bystander activation. Future developments of kinetic models best describing the kinetics of ^89^Zr-Df-Crefmirlimab should include separate cell-trafficking pathways in the model and should be accompanied by longitudinal blood sample collection. Separate studies would be required to quantify the probabilities of ^89^Zr dissociation from the minibody, observing ^89^Zr-labeled metabolites of the tracer, unbinding of the minibody from CD8 receptors, and exocytosis of an internalized minibody tracer in different tissues.

Last, dynamic immunoPET imaging is currently the only available noninvasive technology that can provide in vivo insight into whole-body CD8^+^ T cell distribution and trafficking in human subjects and, although based on a very small group of subjects, appears to offer higher sensitivity than the peripheral blood assays for studying CD8^+^ T cell physiology in individual subjects. The specificity of this and other immunoPET tracers under development, combined with the high detection sensitivity of total-body PET now, provides a new platform for noninvasively and longitudinally studying the immune response and memory in all organs of the body in individual subjects in processes that challenge or stimulate the cell-mediated immune system. This includes, but is not limited to, cancer, infectious disease, autoimmune disease, and transplant patients and can be used for prognosis as well as therapeutic and vaccine developments.

## MATERIALS AND METHODS

A pilot total-body PET imaging study with ^89^Zr-Df-Crefmirlimab was performed. The protocol was approved by the Institutional Review Board (#1692510), and all participants provided written informed consent.

### Study design

The study consisted of two groups, including patients recovering from COVID-19 and healthy control individuals. The COVID-19 convalescent patients had a previous mild or moderate symptomatic infection and were not hospitalized. They all had positive identification of SARS-CoV-2 nucleic acids by a polymerase chain reaction (PCR) assay or SARS-CoV-2 nucleocapsid protein antigen identification at the time of diagnosis. The exclusion criteria for COVID-19 convalescent patients were subjects with serious comorbidities, history of splenic disorders or splenectomy, or use of medications that may affect T cells. Healthy controls gave no history of cancer or autoimmune disease within the last 5 years, no history of immune modulating therapy, no viral infection currently or within the 4 weeks before the study, and no history of COVID-19 infection, which was confirmed by negative detection of immunoglobulin G (IgG) antibodies against the nucleocapsid protein of SARS-CoV-2 in subjects vaccinated against COVID-19 and negative detection of IgG antibodies to the spike protein (S1/S2) of SARS-CoV-2 in unvaccinated subjects. A negative SARS-CoV-2 nucleic acid finding by a PCR assay was required before the first imaging visit for all subjects in both groups.

### Radiotracer formulation and administration

Crefmirlimab-berdoxam was radiolabeled with ^89^Zr at either Optimal Tracers, CA, USA or Memorial Sloan Kettering Cancer Center, NY, USA. Each patient dose contained a ~1.5-mg mass dose of crefmirlimab-berdoxam anti-CD8 minibody (ImaginAb Inc., USA). The radiochemical purity determined by instant thin-layer chromatography was more than 95% in all cases. An ~18.5-MBq (0.5 mCi) dose of ^89^Zr-Df-Crefmirlimab was infused intravenously over 5 to 10 min using a syringe pump, followed by clearance with 30 cc of normal saline. Estimated effective radiation dose was 12 mSv. No premedications were administered, and vital signs were recorded before infusion, after infusion, and at the end of imaging.

### PET/CT imaging

Subjects had total-body PET/CT scans on the uEXPLORER scanner (United Imaging Healthcare, Shanghai, China) at three time points, including a 90-min PET dynamic scan starting immediately before the infusion, followed by two 60-min PET scans at 6 and 48 hours p.i. A low-dose CT scan (dose modulated, max 50 average mAs, 140 kVp, 10-mSv estimated effective radiation dose) was acquired before the first PET scan of each subject, and ultralow-dose CT scans (dose modulated, max 6 average mAs, 140 kVp, 1-mSv estimated effective radiation dose) were acquired before the later time point PET scans. The COVID-19 convalescent patients were first scanned within 8 weeks from onset of their symptoms and returned after 4 ± 1 months for a second set of PET/CT scans.

PET images were reconstructed using the vendor’s image reconstruction software, which used an iterative time-of-flight ordered subset expectation maximization algorithm, with a reconstruction field of view of 60 cm and four iterations (20 subsets). A first set of images were reconstructed at a high spatial resolution using a 512 × 512 matrix with 1.172-mm isotropic voxels and point spread function (PSF) modeling, using 60 min of the listmode data from each time point (30 to90 min from the dynamic scans) and were used for visualization and localization of the volumes of interest (VOIs), particularly for smaller structures such as the lymph nodes and vertebrae. A second set of images were reconstructed at a lower spatial resolution using a 256 × 256 matrix with 2.344-mm isotropic voxels and no PSF modeling, which were used for data analysis in all organs. The dynamic datasets were reconstructed using the latter setting, and 6 × 60–, 16 × 30–, 2 × 60–, 12 × 120–, and 10 × 300–s frames were generated. All corrections recommended by the manufacturer were applied, and no post-reconstruction smoothing filters were used.

### Image analysis

PET/CT images from 60-min reconstructions of all time points were first visualized in AMIDE medical image analysis software (*44*). A qualitative assessment of the tracer distribution was performed at each time point, and ~100 spherical VOIs were drawn on each image over the spleen, bone marrow (vertebrae, sacrum, and ilium), liver, lungs, thymus (when visible in PET), left ventricle (LV) blood pool, right ventricle (RV) blood pool, head and neck lymph nodes, palatine tonsils, cerebrum, cerebellum, and nasal cavity mucosa. For large organs, several small VOIs were placed on the organ, excluding regions that may have been affected by motion and regions in proximity of blood vessels as much as possible. The coordinates and dimensions of the VOIs were transferred to MATLAB R2021b (The MathWorks Inc., Natick, MA, USA), and image analysis was performed using an in-house–developed code package. A segmented organ map was created for each dataset by first calculating the mean and SD of PET voxel values covered by the initial set of small VOIs for each organ and, subsequently, increasing the VOI diameters and assigning an organ index to the voxels with PET values within the mean ± SD of the organ and CT values within a predefined Hounsfield unit range specific to the organ. The SUV—defined as the ratio of image-derived activity concentration (decay corrected to the injection time) to the administered dose divided by the body weight—was expressed as SUV_mean_ for all organs, calculated from the mean of all voxels assigned to the organ, except for the lymph nodes, for which SUVpeak was calculated for each lymph node individually. SUVpeak was defined as the mean value of the eight hottest voxels within the VOI, equivalent of ~0.1-ml volume. Air fraction correction in the lungs was investigated using the coregistered CT image values. Thymus fatty degeneration was evaluated in all subjects using the low-dose CT images, with four-point scores: score 0 representing complete fatty replacement of the thymus and score 3 representing a solid thymic gland with predominantly soft tissue attenuation (*40*). The TAC of image-derived LV blood pool activity concentration was plotted for each dataset and fitted with a triexponential function to derive the whole blood clearance rate.

### Kinetic modeling

TBR was calculated from the ratio of the tissue activity concentration in each organ (*C*_T_) to the whole blood activity concentration (*C*_p_) at any given time. Note that the subscript p in *C*_p_ conventionally represents the plasma activity concentration, whereas in this study, image-derived whole blood activity concentration from the LV blood pool was used for all organs, except for the lungs, for which the RV blood pool was used. Patlak graphs (*45*) were generated by plotting the TBR versus normalized time defined as $\int_{\tau =0}^{t}C_{p}d\tau /C_{p}$, in which triexponential fitted whole blood TACs were used for integration. Three conventional compartmental tracer kinetic models were fitted on each TAC to investigate feasibility of ^89^Zr-immunoPET kinetic modeling and to assess its reliability in representing the underlaying biology in different organs. This included the one-tissue compartmental model with three fitting microparameters (1T3P) of (*v*_b_, *K*_1_, *k*_2_), two-tissue compartmental model with four fitting microparameters (2T4P) of (*v*_b_, *K*_1_, *k*_2_, *k*_3_), and two-tissue compartmental model with five fitting microparameters (2T5P) of (*v*_b_, *K*_1_, *k*_2_, *k*_3_, *k*_4_) (fig. S22). Model fitting was performed in all cases using all available data points up to 49 hours p.i. The Levenberg-Marquardt algorithm was used for nonlinear least squares fitting using nonuniform weighting factors defined for each time frame based on the frame duration and decay factor. Because of higher uncertainties in the two late time points at 6 and 48 hours p.i.—particularly due to significantly higher statistical noise in the input function measurement as a result of blood clearance—and availability of just a single data point at each late time point, the weighting factors were reduced by a factor of 10 for the two late time points. The AIC with a correction for small sample sizes was used as an estimate of the prediction error to choose the model best fitting the data. In case of the 2T model, *K*_i_ was calculated as a macroparameter representing the net influx rate of the tracer, defined as *K*_i_ = *K*_1_*k*_3_/(*k*_2_ + *k*_3_). To determine whether model parameters can be accurately estimated in the presence of noise, practical identifiability analysis was performed in the lungs, spleen, bone marrow (sacrum and ilium selected as regions less prone to motion), tonsils, and selected occipital lymph nodes, including calculation of normalized sensitivity curves, correlation matrix, bias, SD, and root mean square error of the microparameters, as previously described (*46*). The scaling factor used for modeling the TAC noise was calculated for each subject separately by comparing the measured TAC to the modeled TAC, and 100 TACs were simulated with the noise model in each case to calculate the bias and SD of the microparameter estimates.

### Peripheral blood assays

Before the radiotracer infusion, a ~ 20 ml of whole blood sample was drawn intravenously from each subject into vacutainer tubes containing EDTA. Peripheral blood mononuclear cells (PBMCs) were isolated from whole blood by Ficoll-Hypaque (Cytiva, Marlborough, MA, USA) density gradient centrifugation, and red blood cell lysis was performed using ACK lysis buffer (Gibco/Thermo Fisher Scientific, Waltham, MA, USA). PBMCs were viably cryopreserved in fetal calf serum (FCS) with 10% dimethyl sulfoxide (DMSO) and stored at −140°C in liquid nitrogen (LN_2_).

At the conclusion of the study, PBMCs were thawed and rested overnight in R15 [RPMI-1640 supplemented with 15% FCS, penicillin (100 U/ml), streptomycin (100 mg/ml), and 2 mM l-glutamine] at 37°C and 5% CO_2_. Immunophenotyping was performed by first staining the PBMC for dead cells using a fixable amine-reactive viability dye, followed by an extracellular stain using the fluorescently conjugated monoclonal antibodies listed in table S5 in the presence of brilliant stain buffer (BD Bioscience) to reduce aggregate formation. Cells were then fixed in 1% formaldehyde, and flow cytometry was performed within 24 hours. CD8^+^ and CD4^+^ T cells identified from viable CD3^+^ cells were further delineated on the basis of CD45RA and CCR7 expression into memory subsets: central memory, effector memory, terminally differentiated effector memory (TEMRA), or naïve T cells (fig. S23). T cell activation was investigated on the basis of CD56 expression and coexpression of HLA-DR and CD38, and T cell exhaustion was assessed by PD-1 expression (fig. S24). T_reg_ cells (CD4^+^, CD25^+^, and CD127^−^) and MAIT cells (CD161^+^ and Vα7.2^+^) were gated, NK cells were gated on the basis of CD56 and CD16 expression, and B cells were gated on the basis of CD19 (fig. S25).

PBMCs were also assessed for responsiveness to SARS-CoV-2 peptides by intracellular cytokine staining, as previously described (*47*, *48*), using the flow cytometry panel described in table S6. After overnight resting, PBMCs were incubated in R15 in the presence of stimulation cocktail, including CD107a-phycoerythrin-Cy5 (to measure the degranulation response), unlabeled CD28 and CD49d costimulatory antibodies, and the protein transport inhibitors, brefeldin A (MilliporeSigma, St. Louis, MO, USA) and GolgiStop (monensin, BD Biosciences, Franklin Lakes, NJ, USA). Cells were stimulated with SARS-CoV2 peptide pools (15-nucleotide oligomer overlapping by 11) spanning the spike and nucleocapsid proteins (JPT Peptides, Berlin, DE) at a concentration of 3.5 μg/ml for 5 hours at 37°C and 5% CO_2_. DMSO (peptide carrier) served as a negative control, and staphylococcal enterotoxin B (5 μg/ml) served as a positive control. Following stimulation, cells were stained for viability and extracellular markers. They were then fixed in 4% formaldehyde and permeabilized using BD FACS Perm 2 (BD Biosciences), and intracellular staining was performed. Brilliant stain buffer was used during the extracellular and intracellular staining steps to minimize aggregate formation. Samples were then fixed in 1% formaldehyde, and flow cytometry was performed on the next day. Detection of intracellular effector molecules (IFN-γ, IL-2, TNF-α, MIP-1β, and granzyme B) and the degranulation (CD107a) response were measured in memory CD8^+^ and CD4^+^ T cells (fig. S26). The data were analyzed to look at the total response, each of the individual responses, and the polyfunctional response (i.e., production of combinations of the analytes above). A previously described statistical algorithm (*49*), based on the total number of collected events (memory CD8^+^ or CD4^+^) using a Poisson distribution, was used to determine whether stimulated responses differed significantly from unstimulated samples to perform background subtraction.

In all cases, flow cytometry was performed with a five-laser, 40-color Aurora spectral cytometer (Cytek, Fremont, CA, USA), and the data were analyzed using FlowJo software version 10.8.1 (BD Biosciences). Polyfunctionality was mapped in SPICE software version 6.1 (*50*).

### Statistical analysis

Hypothesis testing comparing the two sets of scans from the COVID-19 group to the control group was performed on all datasets in GraphPad Prism version 9.5 using a two-tailed unpaired Mann-Whitney *U* test. Spearman’s rank correlation coefficients were calculated in MATLAB R2021b between the peripheral blood CD8^+^ immunophenotyping results and the TBRs from the 6-hour time point in analyzed tissues of interest. *P* values < 0.05 were set to determine statistical significance, and only *P* values with statistical significance were displayed on the figures. No multiple comparison correction was included.
