# Supplementary material for: First-in-human immunoPET imaging of COVID-19 convalescent patients using dynamic total-body PET and a CD8-targeted minibody
Source: Sci Adv. 2023 Oct 12;9(41):eadh7968. doi: 10.1126/sciadv.adh7968 (PMC10569706; doi:10.1126/sciadv.adh7968)
Supplement: Supplementary file 1 — Figs. S1 to S26 Tables S1 to S6 [file sciadv.adh7968_sm.pdf]

Science Advances
AAAS

Supplementary Materials for

First-in-human immunoPET imaging of COVID-19 convalescent patients using dynamic total-body PET and a CD8-targeted minibody

Negar Omidvari *et al.*

Corresponding author: Negar Omidvari, nomidvari@ucdavis.edu

Sci. Adv. **9**, eadh7968 (2023)
DOI: 10.1126/sciadv.adh7968

This PDF file includes:

Figs. S1 to S26
Tables S1 to S6

SUPPLEMENTARY MATERIALS

Table S1. Complete demographics of the participants. Vaccination and diagnosis timelines are included. Note: No patient recorded central nervous system or cardiac symptoms of COVID-19.

Subject number	Study group	Sex	Age	BMI	Dynamic scan	Vaccination and diagnosis Timeline
1	COVID-19	F	51	38	-	Infected with COVID-19 in March 2021 before vaccination and was sick for 3-4 weeks.<br>Symptoms: shortness of breath, abdominal pain, nausea, mild headache<br>First PET scan was 11 days after the first vaccination dose and second PET scan was 3 months after the second vaccination dose.<br>Past medical history: Cushing disease (pituitary removed in 2012), diabetes, hyperlipidemia, and hypothyroid
2	COVID-19	F	29	29	90 min	Infected with COVID-19 in January 2021 (asymptomatic), 13 days after the first vaccination dose. Infected with COVID-19 again in April 2021, 3 months after the second dose of vaccination and was sick for 2 weeks.<br>Symptoms: loss of taste and smell, fatigue, difficulty breathing, headaches, nausea, congestion, sore throat, runny nose, extreme stomach pain after eating, lightheadedness, extreme exhaustion, shortness of breath, extreme body aches<br>First PET scan was 4 months after the second vaccination dose and second PET scan was 8.5 months after the second vaccination dose.<br>Past medical history: gastric bypass, iron deficiency anemia, back pain with radiculopathy, laminectomy
3	COVID-19	F	46	43	-	Infected with COVID-19 in May 2021, 3.5 months after the second vaccination dose and was sick for 1 week.<br>Symptoms: chronic cough, headache, sore throat, fever, chills, abdominal pain, congestion, body aches, dysuria, muscle pain<br>First PET scan was 4.5 months after the second vaccination dose and second PET scan was 8.5 months after the second vaccination dose.<br>Past medical history: cholecystectomy, sleeve gastrectomy, tubal ligation
4	COVID-19	F	27	35	90 min	Infected with COVID-19 in February 2022, 3 months after the third vaccination dose and was sick mildly for 5-6 days.<br>Symptoms: cough, diarrhea, fatigue, fever, slight headache, sore throat<br>First PET scan was 4.5 months after the second vaccination dose and second PET scan was 7 months after the third vaccination dose.<br>Past medical history: none
5	COVID-19	F	34	20	90 min	Infected with COVID-19 in March 2022, 8 months after the second vaccination dose and was mildly sick for 3 days.<br>Symptoms: cough, fatigue, congestion, runny nose<br>First PET scan was 9.5 months after the second vaccination dose and second PET scan was 4 months after the third vaccination dose.<br>Past medical history: cervical polypectomy.
6	Control	M	25	21	90 min	First PET scan was 6 months after the second vaccination dose.<br>Past medical history: not available.
7	Control	M	49	25	65 min	First PET scan was 6 months after the second vaccination dose.<br>Past medical history: femoral bone fracture, hypertension.
8	Control	F	59	31	90 min	Not vaccinated.<br>Past medical history: not available.

Table S2. Microparameters of model fits in all organs-of-interest of individual subjects. Microparameters of AIC-preferred model fit results for lungs, spleen, sacrum, ilium, tonsils, and occipital lymph nodes shown for all subjects with dynamic scans. K_1 is shown in $ml_{plasma}/min/ml_{tissue}$, and k_2, k_3, and k_4 are shown in 1/min. *Ki* is calculated by $\frac{K_1 k_3}{k_2+k_3}$ and V_T is calculated by $\frac{K_1}{k_2}\left(1+\frac{k_3}{k_4}\right)$.

Lungs - 2T5P

	v_b	K_1	k_2	k_3	k_4	K_i	V_T
Sub02 - Baseline	0.22	0.02	0.16	0.004	0.0013	0.00054	0.56
Sub02 – Follow-up	0.24	0.03	0.18	0.017	0.0074	0.00239	0.50
Sub04 - Baseline	0.17	0.00	1.00	0.050	0.0006	0.00018	0.31
Sub04 - Follow-up	0.14	0.05	0.44	0.001	0.0003	0.00014	0.56
Sub05 - Baseline	0.17	0.01	0.58	0.003	0.0000	0.00005	-
Sub05 - Follow-up	0.16	0.00	0.04	0.009	0.0005	0.00012	0.30
Sub06	0.13	0.02	0.50	0.001	0.0000	0.00004	-
Sub07	0.23	0.01	0.52	0.000	0.0029	0.00000	0.01
Sub08	0.18	0.02	0.32	0.002	0.0001	0.00012	1.28

Spleen - 2T5P

	v_b	K_1	k_2	k_3	k_4	K_i	V_T
Sub02 - Baseline	0.40	1.48	0.04	0.0040	0.0015	0.12	124.4
Sub02 – Follow-up	0.40	1.43	0.04	0.0049	0.0021	0.17	128.5
Sub04 - Baseline	0.40	0.88	0.06	0.0037	0.0007	0.05	86.8
Sub04 - Follow-up	0.40	1.05	0.06	0.0047	0.0009	0.07	101.8
Sub05 - Baseline	0.40	1.08	0.08	0.0028	0.0005	0.04	87.0
Sub05 - Follow-up	0.40	0.88	0.07	0.0030	0.0005	0.03	88.2
Sub06	0.40	0.84	0.07	0.0026	0.0004	0.03	87.0
Sub07	0.40	0.85	0.07	0.0018	0.0003	0.02	99.0
Sub08	0.40	1.09	0.06	0.0033	0.0007	0.05	97.6

Sacrum - 2T5P

	v_b	K_1	k_2	k_3	k_4	K_i	V_T
Sub02 - Baseline	0.06	0.08	0.02	0.0041	0.0026	0.017	13.1
Sub02 – Follow-up	0.08	0.09	0.01	0.0033	0.0036	0.022	16.9
Sub04 - Baseline	0.06	0.04	0.02	0.0027	0.0008	0.004	8.3
Sub04 - Follow-up	0.02	0.04	0.02	0.0046	0.0012	0.007	8.9
Sub05 - Baseline	0.02	0.09	0.04	0.0015	0.0005	0.003	8.9
Sub05 - Follow-up	0.05	0.08	0.04	0.0015	0.0004	0.003	10.8
Sub06	0.04	0.05	0.04	0.0019	0.0005	0.002	5.5
Sub07	0.03	0.05	0.03	0.0019	0.0003	0.003	11.8
Sub08	0.03	0.03	0.02	0.0008	0.0001	0.001	9.9

Ilium - 2T5P

	v_b	K_1	k_2	k_3	k_4	K_i	V_T
Sub02 - Baseline	0.05	0.08	0.02	0.0039	0.0026	0.015	12.0
Sub02 – Follow-up	0.08	0.08	0.01	0.0032	0.0038	0.020	16.0
Sub04 - Baseline	0.00	0.05	0.02	0.0025	0.0008	0.005	8.3
Sub04 - Follow-up	0.03	0.04	0.01	0.0017	0.0007	0.005	10.5
Sub05 - Baseline	0.02	0.08	0.04	0.0014	0.0004	0.003	8.9
Sub05 - Follow-up	0.03	0.07	0.04	0.0018	0.0005	0.003	9.2
Sub06	0.02	0.04	0.04	0.0015	0.0005	0.002	5.1
Sub07	0.04	0.04	0.02	0.0015	0.0002	0.002	10.8
Sub08	0.00	0.03	0.02	0.0013	0.0005	0.002	5.1

Tonsils - 1T3P

	v_b	K_1	k_2	K_1/k_2
Sub02 - Baseline	0.00	0.014	0.00053	25.9
Sub02 – Follow-up	0.00	0.012	0.00036	32.6
Sub04 - Baseline	0.00	0.004	0.00018	22.3
Sub04 - Follow-up	0.06	0.005	0.00012	42.0
Sub05 - Baseline	0.04	0.005	0.00023	22.7
Sub05 - Follow-up	0.03	0.004	0.00018	22.5
Sub06	0.05	0.004	0.00014	30.2
Sub07	0.05	0.001	0.00000	-
Sub08	0.05	0.002	0.00000	-

Occipital Lymph Nodes - 1T3P

	v_b	K_1	k_2	K_1/k_2
Sub02 - Baseline	0.02	0.0009	0.00040	2.2
Sub02 – Follow-up	0.02	0.0010	0.00092	1.0
Sub04 - Baseline	0.05	0.0012	0.00000	-
Sub04 - Follow-up	0.05	0.0017	0.00000	-
Sub05 - Baseline	0.05	0.0020	0.00000	-
Sub05 - Follow-up	0.05	0.0020	0.00000	-
Sub06	0.06	0.0002	0.00019	1.2
Sub07	0.14	0.0005	0.00000	-
Sub08	0.00	0.0021	0.00024	8.9

Table S3. Correlation matrix of model microparameters. Correlation matrix of microparameters of the AIC-preferred model is presented for lungs, spleen, sacrum, ilium, tonsils, and occipital lymph nodes, showing mean and standard deviations calculated over all subjects with 90-min dynamic scans.

Lungs

	v_b	K_1	k_2	k_3	k_4
v_b	1.00	-0.13 ± 0.64	0.30 ± 0.53	-0.81 ± 0.20	0.71 ± 0.10
K_1		1.00	-0.95 ± 0.05	0.51 ± 0.51	-0.31 ± 0.43
k_2			1.00	-0.61 ± 0.46	0.39 ± 0.39
k_3				1.00	-0.80 ± 0.08
k_4					1.00

Spleen

	v_b	K_1	k_2	k_3	k_4
v_b	1.00	-1.00 ± 0.00	0.96 ± 0.04	-0.79 ± 0.06	0.33± 0.07
K_1		1.00	-0.95 ± 0.04	0.76 ± 0.06	-0.28 ± 0.07
k_2			1.00	-0.86 ± 0.02	0.43 ± 0.09
k_3				1.00	0.75 ± 0.05
k_4					1.00

Sacrum

	v_b	K_1	k_2	k_3	k_4
v_b	1.00	-0.86 ± 0.09	0.92 ± 0.05	-0.81 ± 0.08	0.48 ± 0.06
K_1		1.00	-0.91 ± 0.05	0.55 ± 0.16	-0.12 ± 0.14
k_2			1.00	-0.75 ± 0.18	0.37 ± 0.25
k_3				1.00	-0.81 ± 0.07
k_4					1.00

Ilium

	v_b	K_1	k_2	k_3	k_4
v_b	1.00	-0.86 ± 0.10	0.91 ± 0.05	-0.79 ± 0.07	0.46 ± 0.07
K_1		1.00	-0.91 ± 0.05	0.52 ± 0.09	-0.08 ± 0.15
k_2			1.00	-0.74 ± 0.15	0.34 ± 0.25
k_3				1.00	-0.81 ± 0.06
k_4					1.00

Tonsils

	v_b	K_1	k_2
v_b	1.00	-0.99 ± 0.00	0.85 ± 0.02
K_1		1.00	-0.87 ± 0.03
k_2			1.00

Occipital Lymph Nodes

	v_b	K_1	k_2
v_b	1.00	-0.93 ± 0.07	0.79 ± 0.09
K_1		1.00	-0.88± 0.03
k_2			1.00

Table S4. Estimated errors of model microparameters. Bias (%), standard deviation (%), and RMSE (%) of the AIC-preferred model microparameters in lungs, spleen, sacrum, ilium, tonsils, and cervical lymph nodes, calculated from model fitting on 100 simulated TACs generated for each subject from the calculated noise model of the measured TAC. The results show the averaged values among all subjects with 90-min dynamic scans.

Bias (%)

	Lungs	Spleen	Sacrum	Ilium	Tonsils	Occipital lymph nodes
v_b	2.9	0.7	1.0	-0.4	1.6	7.9
K_1	-9.8	5.6	-0.1	-0.2	0.2	0.8
k_2	-9.0	-0.1	-0.2	-0.5	-0.7	-1.9
k_3	59.8	0.1	-0.3	-1.0		
k_4	26.2	0.1	-0.6	-1.2		

Standard deviation (%)

	Lungs	Spleen	Sacrum	Ilium	Tonsils	Occipital lymph nodes
v_b	6.7	34.3	29.0	40.5	17.2	18.7
K_1	77.4	22.3	1.8	2.3	2.3	7.2
k_2	63.9	4.4	4.7	6.2	7.7	22.4
k_3	113.8	3.1	6.4	10.5		
k_4	197.3	2.7	7.0	7.0		

RMSE (%)

	Lungs	Spleen	Sacrum	Ilium	Tonsils	Occipital lymph nodes
v_b	7.5	34.3	29.2	40.4	17.3	20.8
K_1	82.8	23.1	1.8	2.3	2.3	7.4
k_2	67.7	4.4	4.7	6.2	8.9	22.4
k_3	132.5	3.1	6.4	10.5		
k_4	200.4	2.7	7.0	7.1		

Table S5. Flow cytometry panel design used for cell immunophenotyping. All antibodies and viability dyes were titrated to determine the optimal concentration for each assay.

Antibody/Reagent	Clone	Fluorochrome	Producer	Significance
Viability	--	Live/Dead Blue	ThermoFisher Scientific	Live/Dead
CD3	UCHT1	Alexa Fluor 532	ThermoFisher Scientific	T cell marker
CD4	RPA-T4	APC-Fire750	Biolegend	"Helper" T cells
CD8	SK1	BUV805	BD Biosciences	"Cytotoxic" T cells
CD16	3G8	BV480	BD Biosciences	FcγRIII, NK
CD56	HCD56	BV650	Biolegend	NK, activated CD8
Vα7.2	3C10	PE-Cy7	Biolegend	MAIT
CD161	DX12	BV605	BD Biosciences	NK, MAIT
CD25	M-A251	PE	Biolegend	IL-2R, Treg
CD127	A019D5	BV750	Biolegend	IL-7Rα
CD19	HIB19	FITC	BD Biosciences	B-cells
CD45RA	HI100	BUV395	BD Biosciences	Naïve/TEMRA T cells
CCR7	3D12	BV421	BD Biosciences	Naïve/Central Memory T cells
CD38	HIT2	PE-CF594	BD Biosciences	Activation marker (with HLA-DR)
HLA-DR	TU36	PerCP-Cy5.5	Biolegend	MHC-II; Activation marker (with CD38)
PD-1	J105	APC	ThermoFisher Scientific	T-cell exhaustion

Table S6. Flow cytometry panel design used for intracellular cytokine staining assays. All antibodies and viability dyes were titrated to determine the optimal concentration for each assay.

Antigen/Marker	Clone	Fluorochrome	Producer	Significance
Viability	--	Live/Dead Aqua	ThermoFisher Scientific	
CD3	UCHT1	Alexa Fluor 532	ThermoFisher Scientific	T cell marker
CD4	SFCI12T4D11	PE-Cy7	Beckman Coulter	"Helper" T-cells
CD8	SK1	BUV805	BD Biosciences	"Cytotoxic" T-cells
CD45RA	HI100	BUV395	BD Biosciences	Naïve/TEMRA
CCR7	3D12	BV421	BD Biosciences	Naïve/Central Memory
CD107a	H4A3	PE-Cy5	BD Biosciences	Cytotoxic granules
CD28	L293	--	BD Biosciences	Costimulatory
CD49d	L25	--	BD Biosciences	Costimulatory
IFNγ	25723.11	APC	BD Biosciences	Proinflammatory
IL2	MQ1-17H12	BV605	BD Biosciences	T-cell growth
MIP-1β	D21-1351	PE	BD Biosciences	Chemotactic
TNFα	MAb11	BV711	Biolegend	Proinflammatory
Granzyme B	GB11	Alexa Fluor700	BD Biosciences	Cytotoxic granules

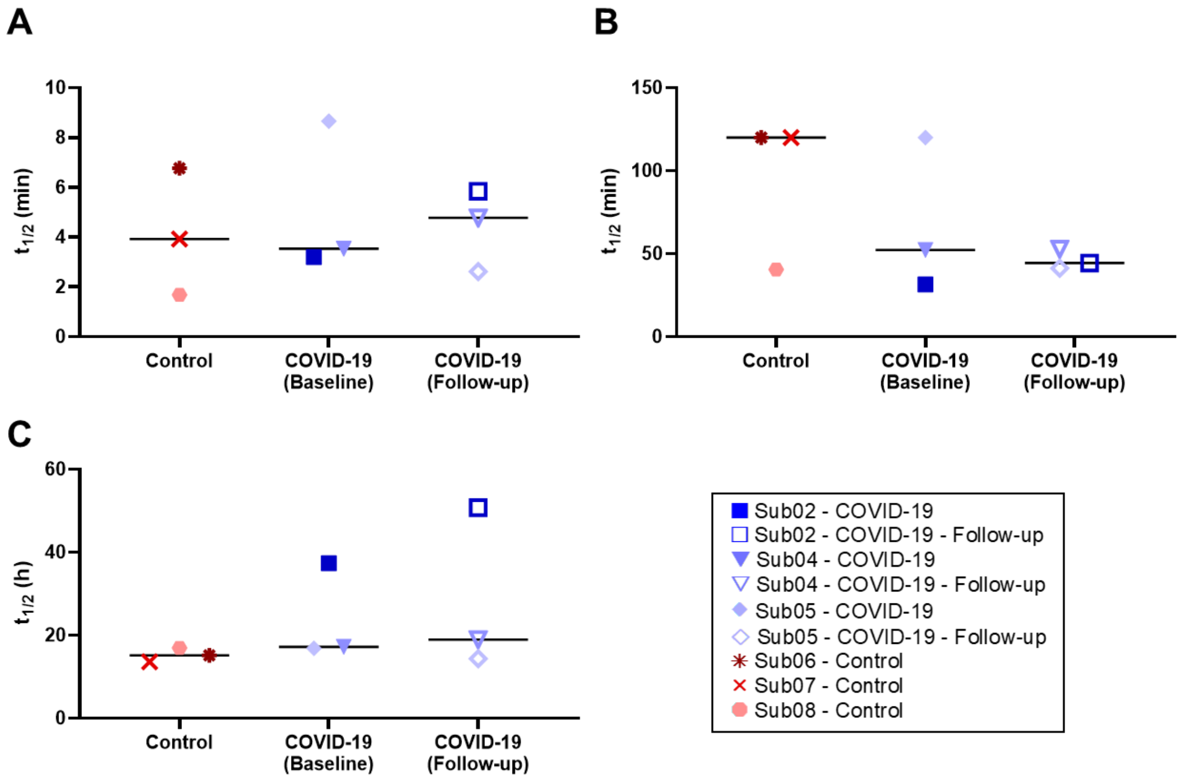

Fig. S1. Whole-blood clearance biological half-lives. Biological half-lives derived from triexponential fitting on the whole-blood TACs of 6 subjects (9 scans), including (**A**) the initial, (**B**) the intermediate, and (**C**) the terminal elimination phases. Image-derived LV blood pool was used for obtaining the whole-blood TACs. With a very low uptake in the myocardium, negligible effects from myocardium spill-over on LV blood pool were observed.

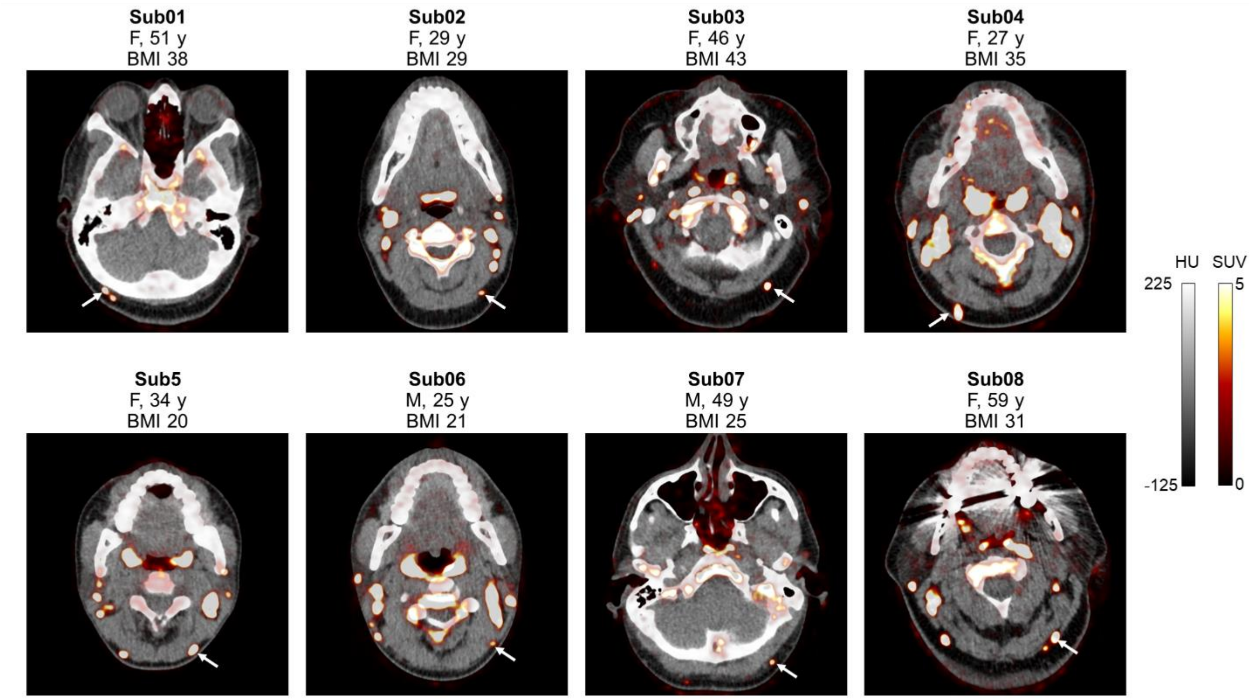

Fig. S2. PET/CT images of selected occipital lymph nodes. Transverse PET/CT slices through representative occipital lymph nodes selected in each subject (marked with arrows), which have been used for kinetic modeling. For COVID-19 patients (Sub01 to Sub05), only the baseline scans are shown.

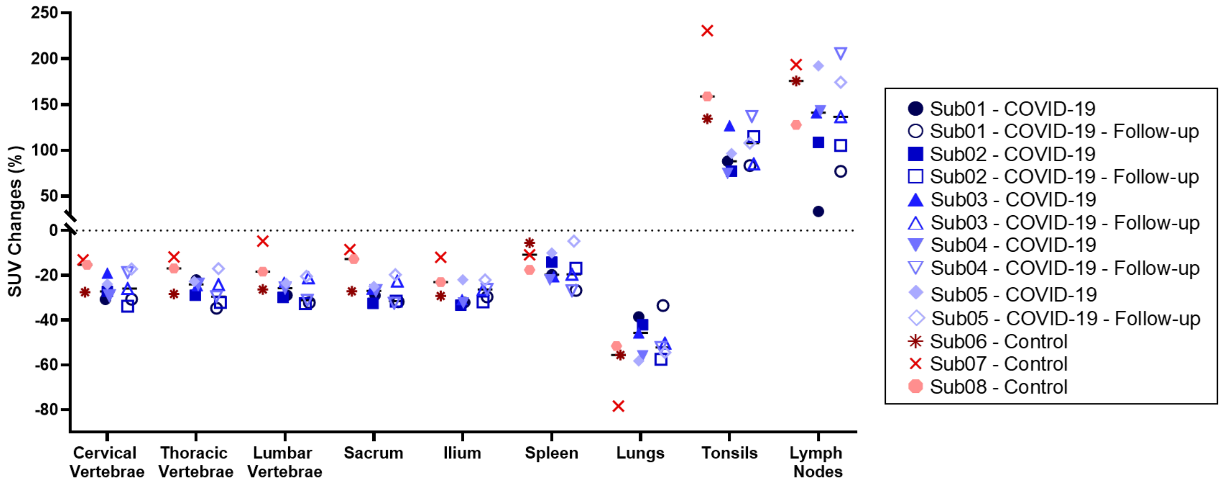

Fig. S3. Percentage changes of SUV at the 48-h relative to the 6-h timepoint. Percentage changes of SUV at the 48-h relative to the 6-h timepoint show similar trends in all subjects, in different regions of bone marrow (vertebrae, sacrum, and ilium), spleen, tonsils, and lymph nodes. The percentage changes of SUV in the lymph nodes represent the average SUV changes in 11–22 head and neck lymph nodes for each subject.

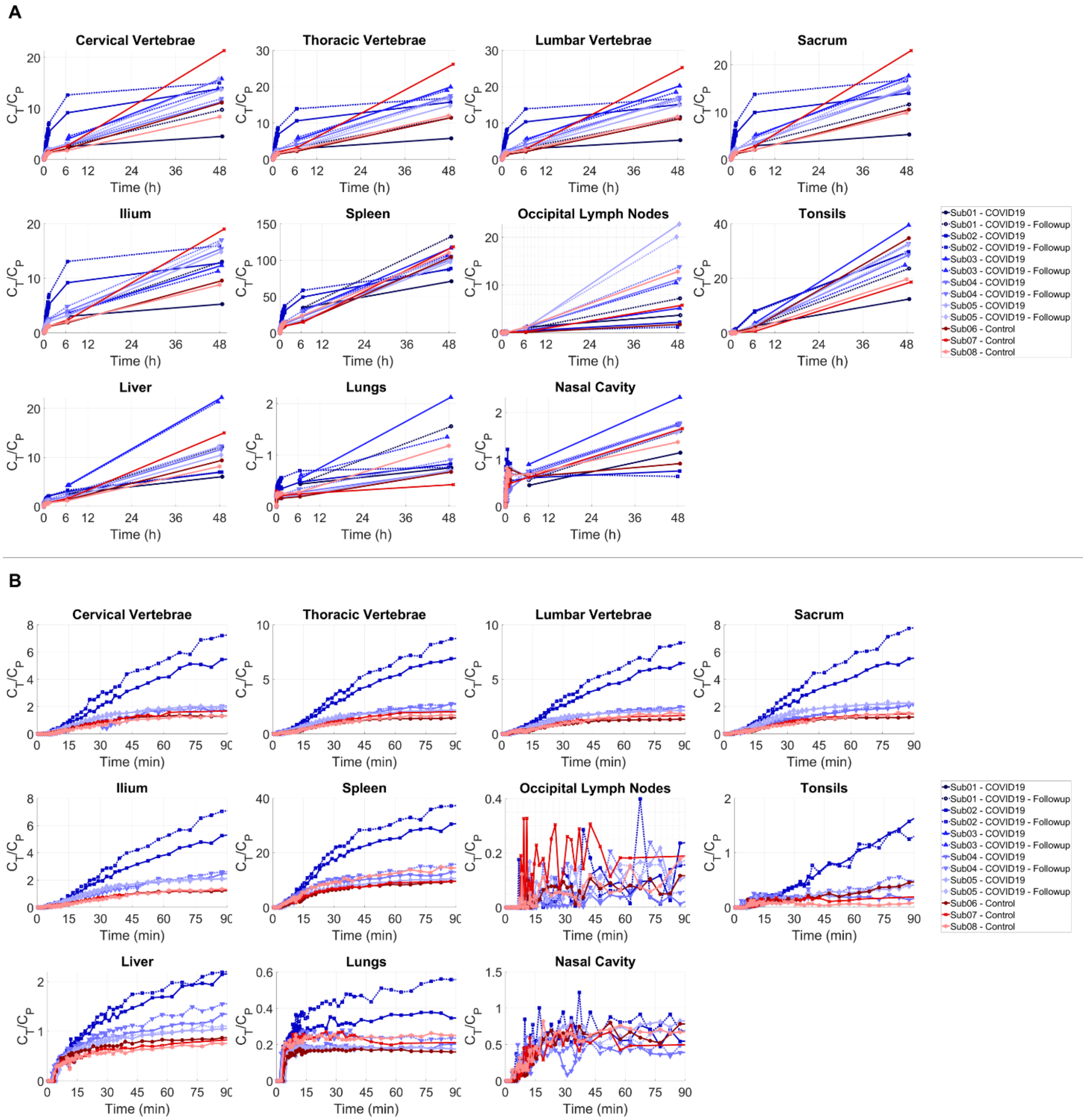

Fig. S4. Decay-corrected TBR curves in different organs-of-interest. TBRs are shown as a function of time for bone marrow, spleen, occipital lymph nodes, tonsils, liver, lungs, and nasal cavity (**A**) during the 48-h of the imaging study and (**B**) during the first 90-min after tracer administration for all subjects.

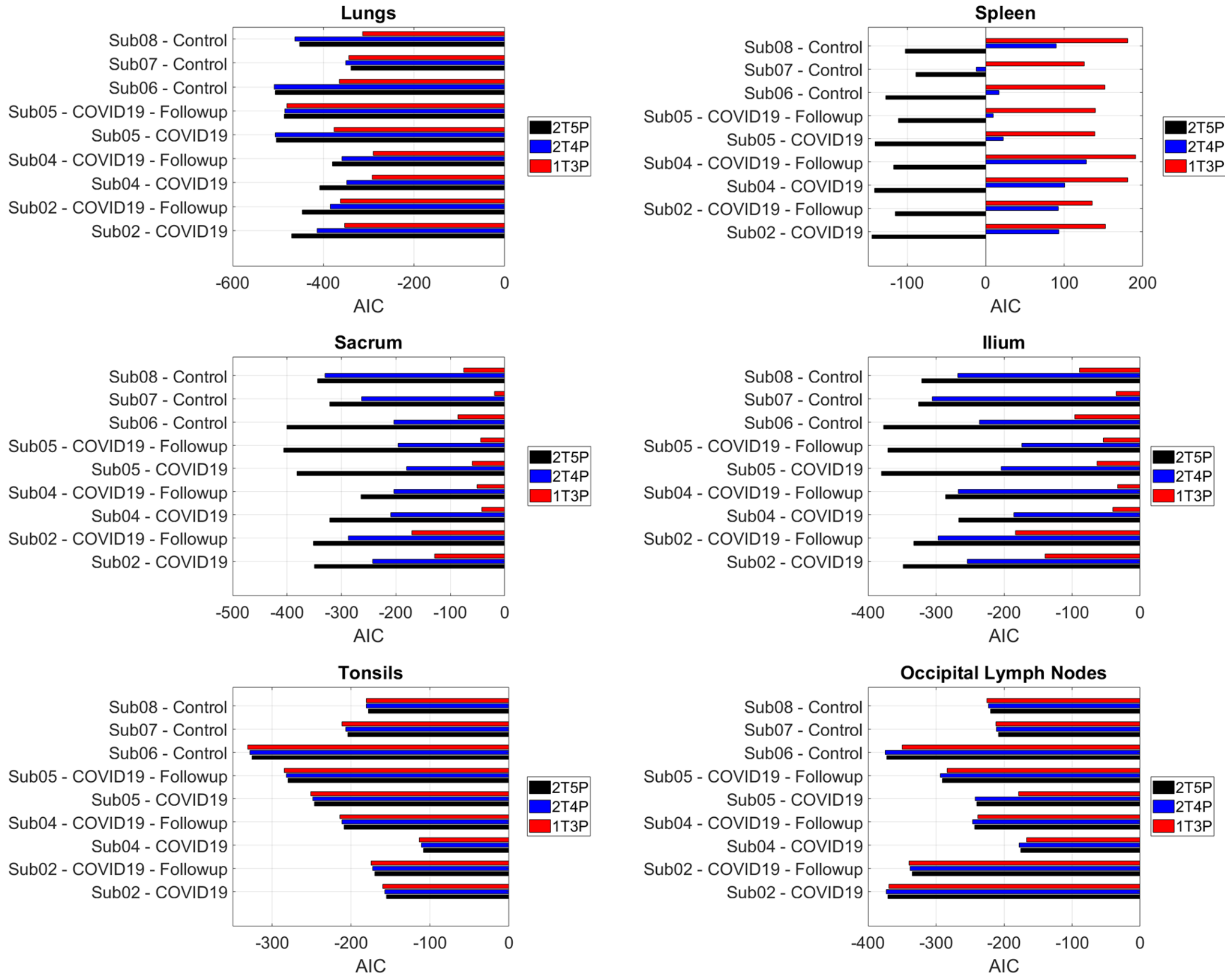

Fig. S5. AIC model selection results in all organs-of-interest. AIC values of 1T3P, 2T4P, and 2T5P model fittings performed on the lungs, spleen, bone marrow (sacrum and ilium), tonsils, and selected occipital lymph nodes of all subjects.

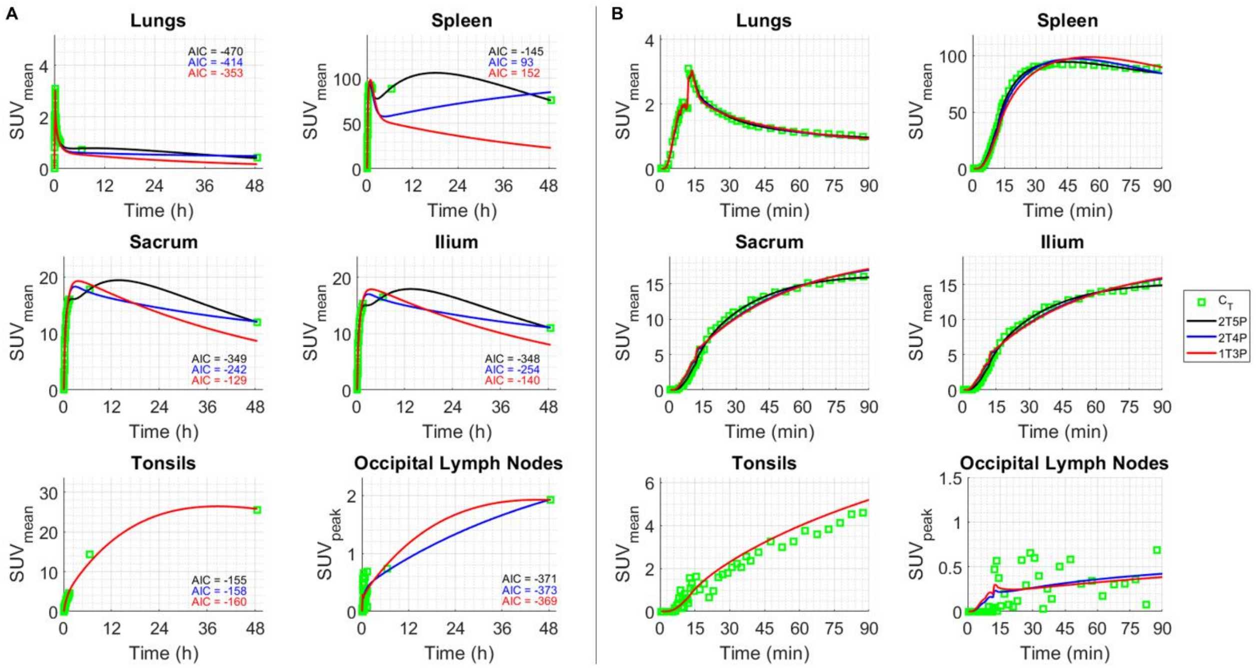

Fig. S6. Examples of model fits in a COVID-19 convalescent subjects. Results of 1T3P, 2T4P, and 2T5P model fittings performed on the lungs, spleen, bone marrow (sacrum and ilium), tonsils, and selected occipital lymph nodes of the baseline scans of an example COVID-19 patient (Sub02) shown for (**A**) the 0–48 h and (**B**) zoomed on the 0–90 min.

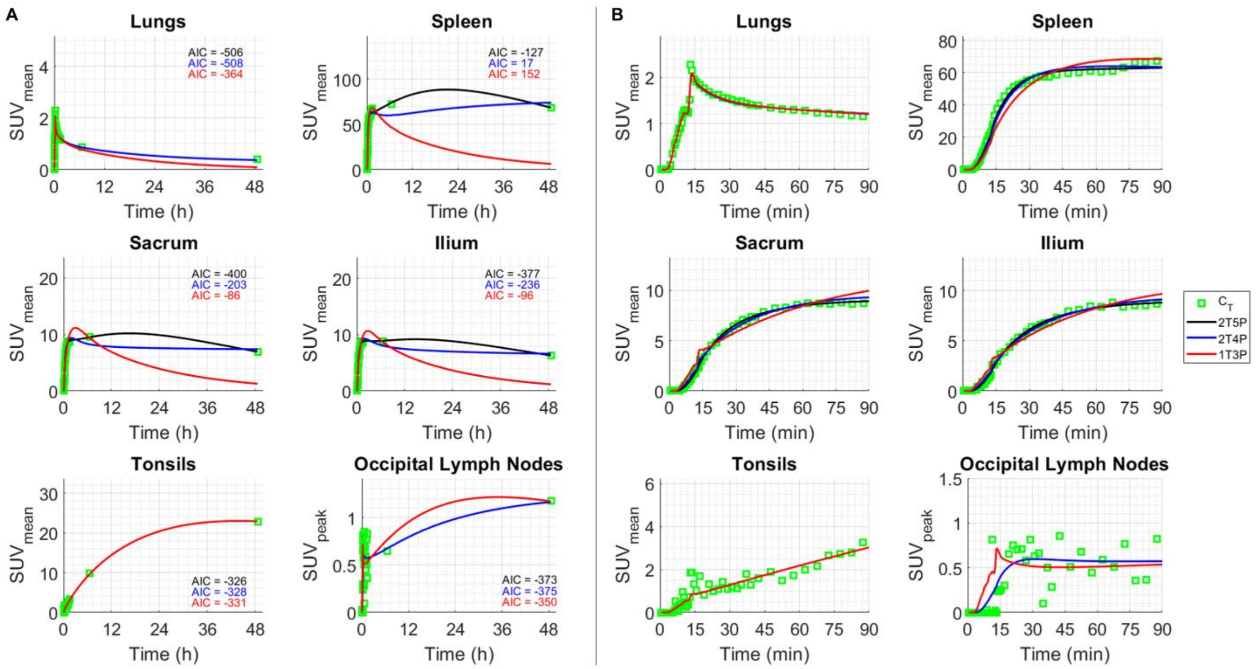

Fig. S7. Examples of model fits in a healthy control subject. Results of 1T3P, 2T4P, and 2T5P model fittings performed on the lungs, spleen, bone marrow (sacrum and ilium), tonsils, and selected occipital lymph nodes of an example control subject (Sub06) shown for (**A**) the 0–48 h and (**B**) zoomed on the 0–90 min.

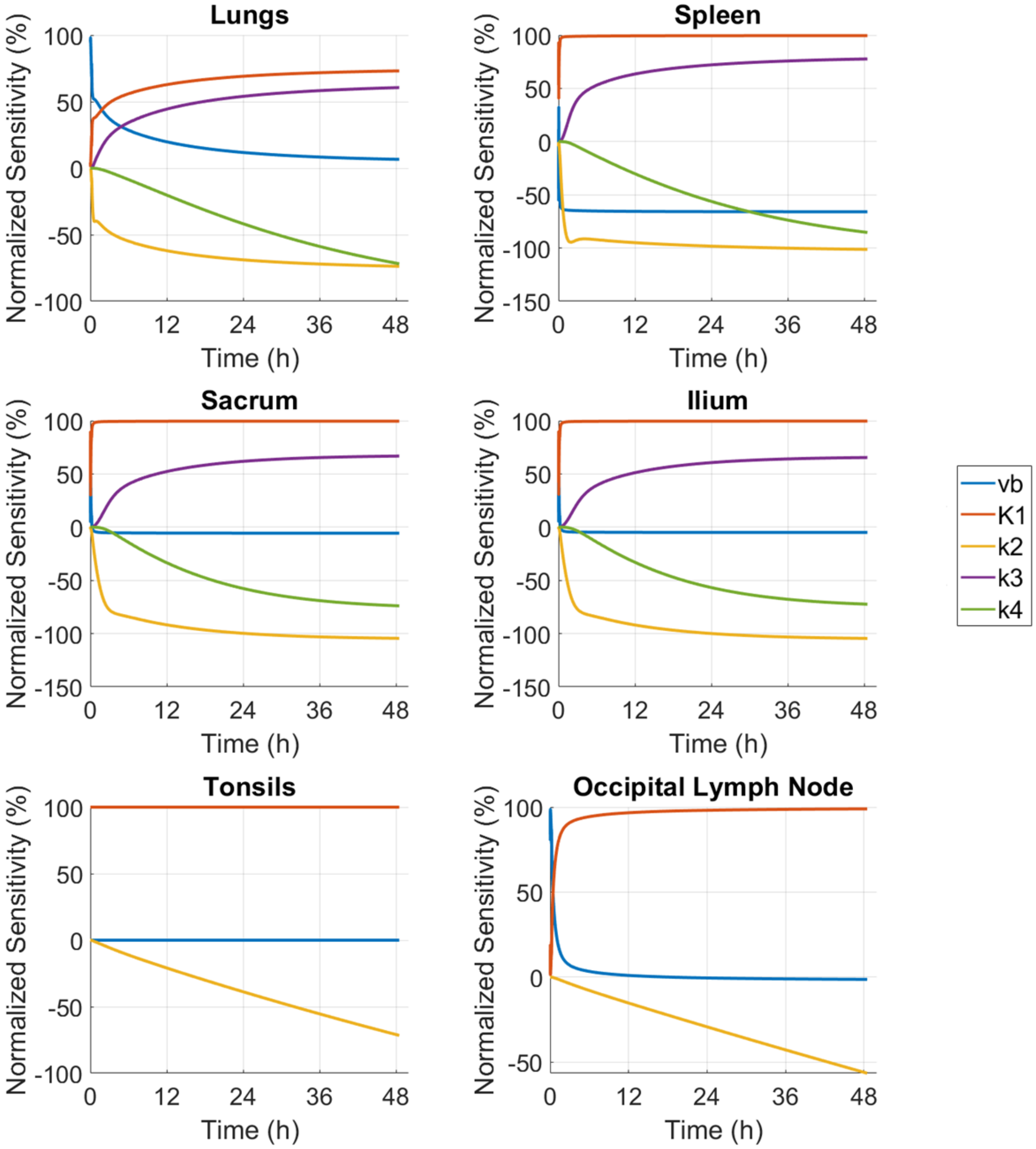

Fig. S8. Normalized sensitivity plots of microparameters of the AIC-preferred model. Normalized sensitivities of microparameters of the model with average highest AIC, shown for lungs, spleen, sacrum, ilium, tonsils, and occipital lymph node of the baseline scans of an example COVID-19 patient (Sub02) shown for the 0–48 h.

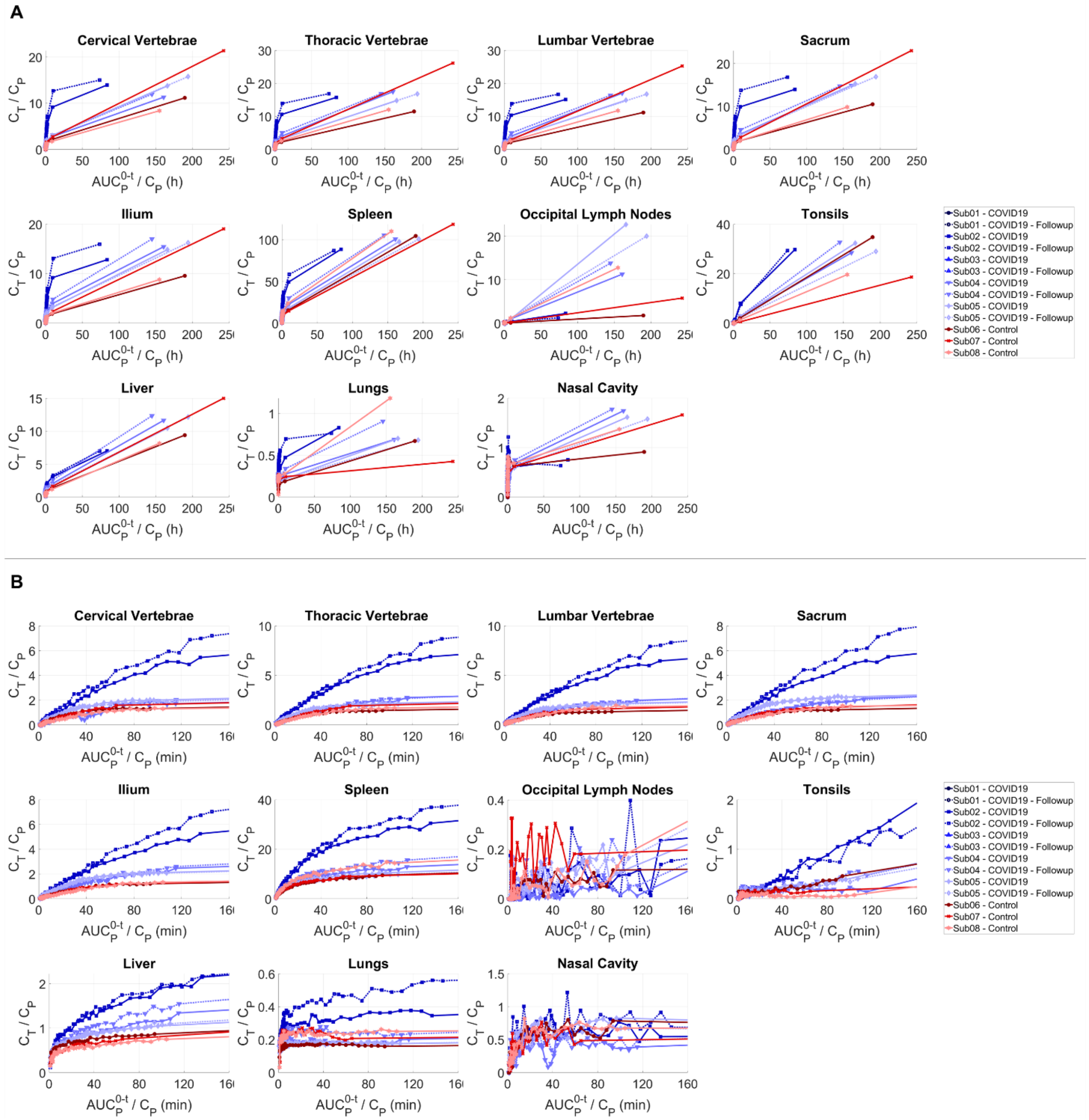

Fig. S9. Patlak plots of different organs-of-interest. Patlak plots of different regions of bone marrow, spleen, occipital lymph nodes, tonsils, liver, lungs, and nasal cavity shown (**A**) during the 48-h of the imaging study and (**B**) during the first 90-min after tracer administration for all subjects with dynamic scans.

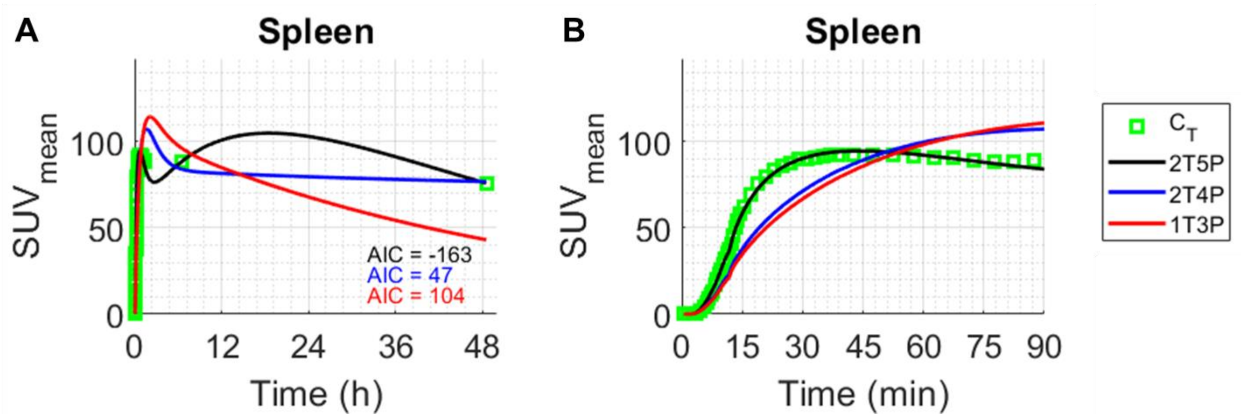

Fig. S10. Effect of late-timepoint weighting factors on model fitting in spleen. Results of 1T3P, 2T4P, and 2T5P model fittings performed on spleen of the baseline scans of an example COVID-19 patient (Sub02) shown for (**A**) the 0–48 h and (**B**) zoomed on the 0–90 min, using increased weighting factors (×10 compared to previous fits) for the two late timepoint scans.

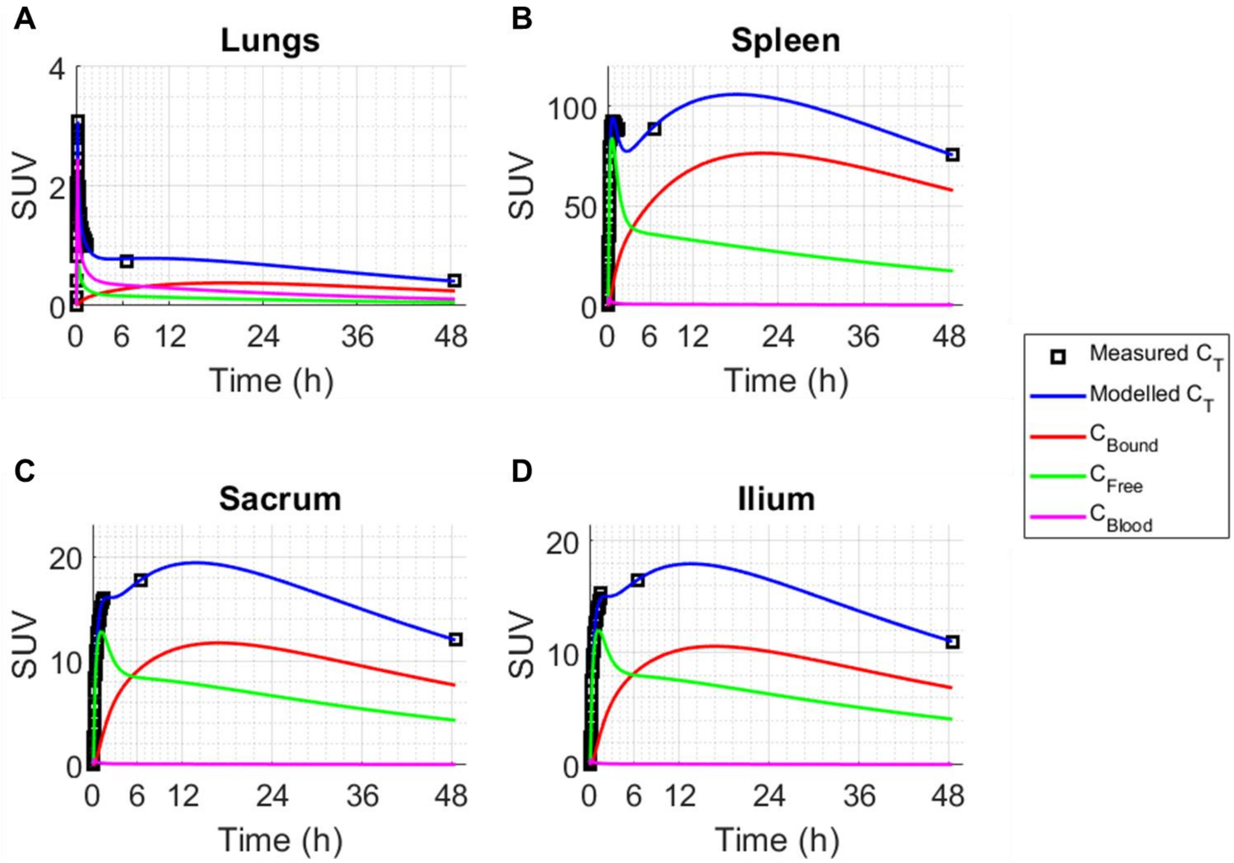

Fig. S11. Changes in concentrations of 2T5P model compartments as a function of time. Concentrations of the compartments of the 2T5P model fit performed on (**A**) lungs, (**B**) spleen and bone marrow ((**C**) sacrum and (**D**) ilium) of the baseline scans of an example COVID-19 patient (Sub02) shown for the 0–48 h of the imaging, including the concentrations of free tracer in tissue (C_{Free}), bound tracer in tissue (C_{Bound}) and the tissue blood fraction (C_{Blood}), in which $C_T = C_{Free} + C_{Bound} + C_{Blood}$.

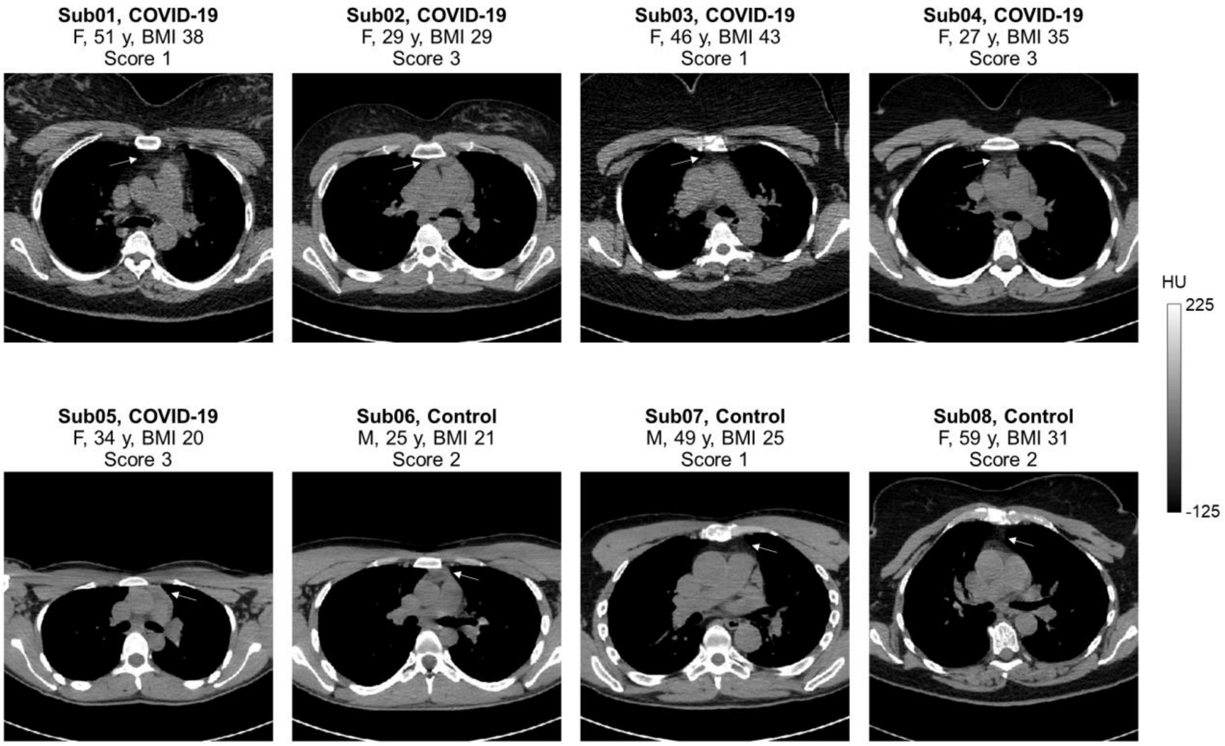

Fig. S12. CT image slices through the thymus of all subjects. Selected slices through low-dose CT images of all subjects are compared with the corresponding thymus fatty degeneration scores assigned to each subject.

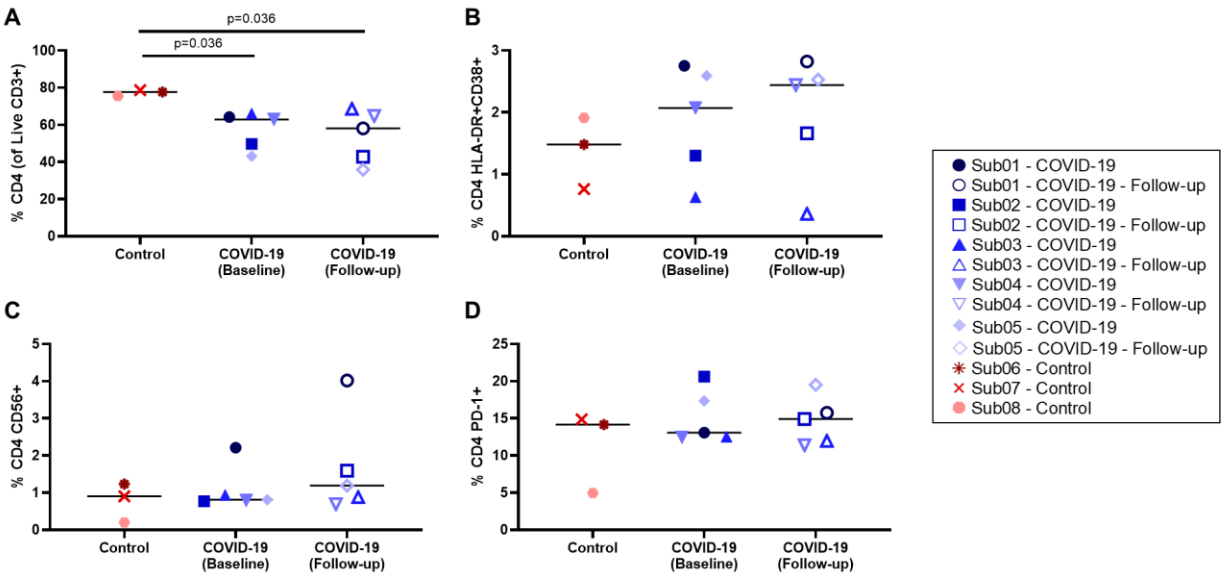

Fig. S13. Peripheral blood CD4$^+$ T cell phenotyping. (**A**) Percentage of CD4$^+$ T cells within the live CD3$^+$ population, (**B**) percentage of activated CD4$^+$ T cells characterized by CD38 and HLA-DR co-expression and (**C**) CD56 expression, and (**D**) percentage of exhausted CD4$^+$ T cells characterized by PD-1 expression in all subjects.

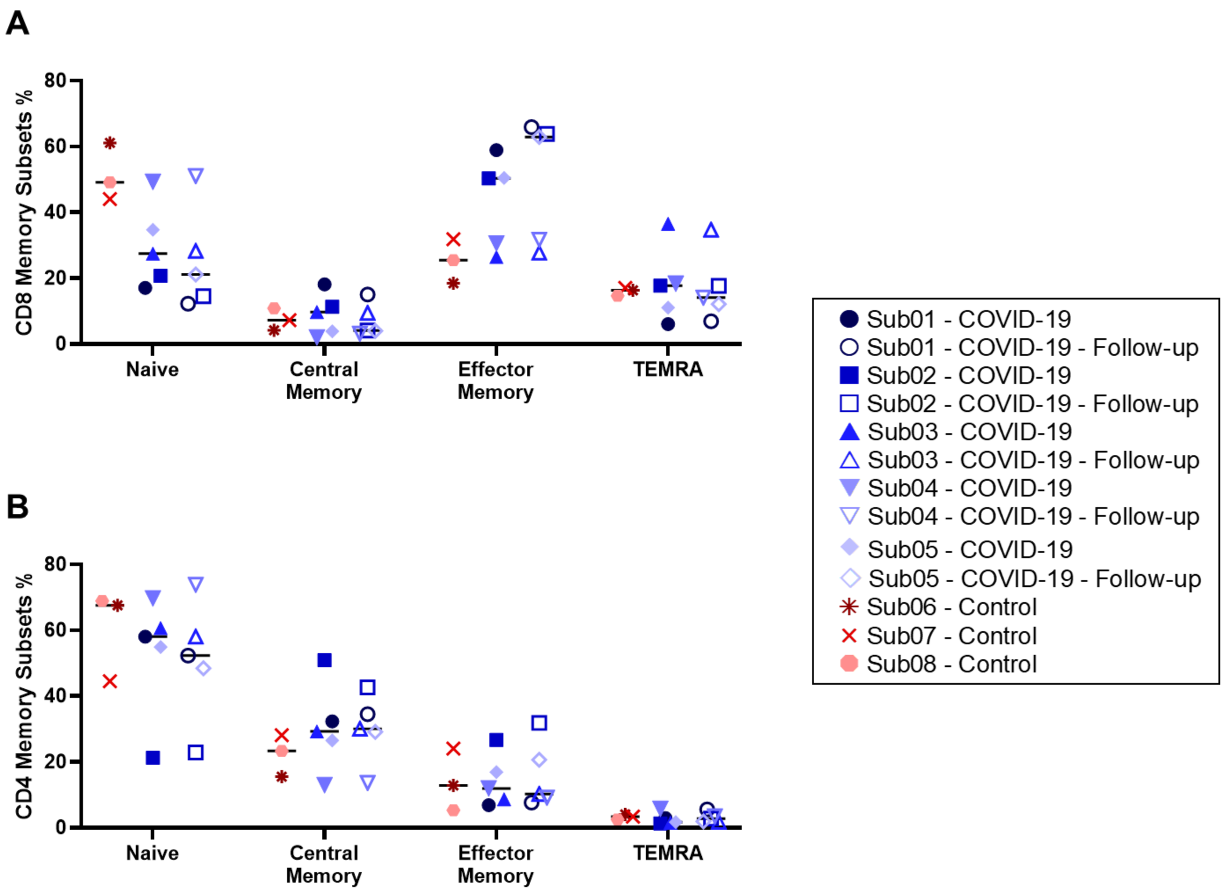

Fig. S14. Memory subsets of CD8$^+$ and CD4$^+$ T cells. Memory subsets of (**A**) CD8$^+$ and (**B**) CD4$^+$ T cells, comparing the percentage of naïve, central memory, effector memory, and TEMRA cells in peripheral blood of all subjects.

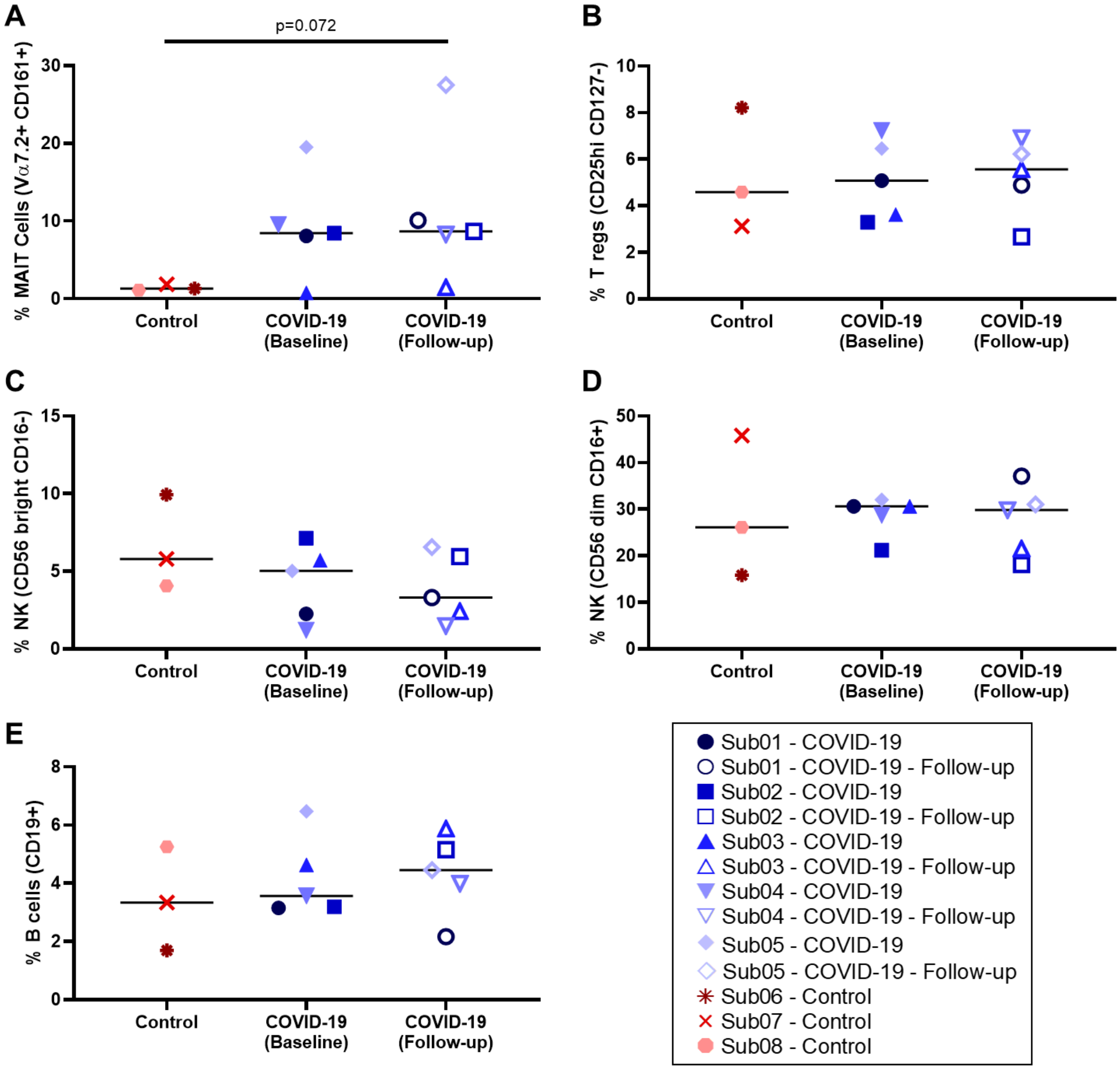

Fig. S15. Peripheral blood phenotyping of MAIT, T_{reg}, NK, and B cells. Percentages of (**A**) MAIT cells, (**B**) T_{reg} cells, (**C**) cytokine-producing NK cells, (**D**) cytotoxic NK cells, and (**E**) B cells compared in peripheral blood of all subjects.

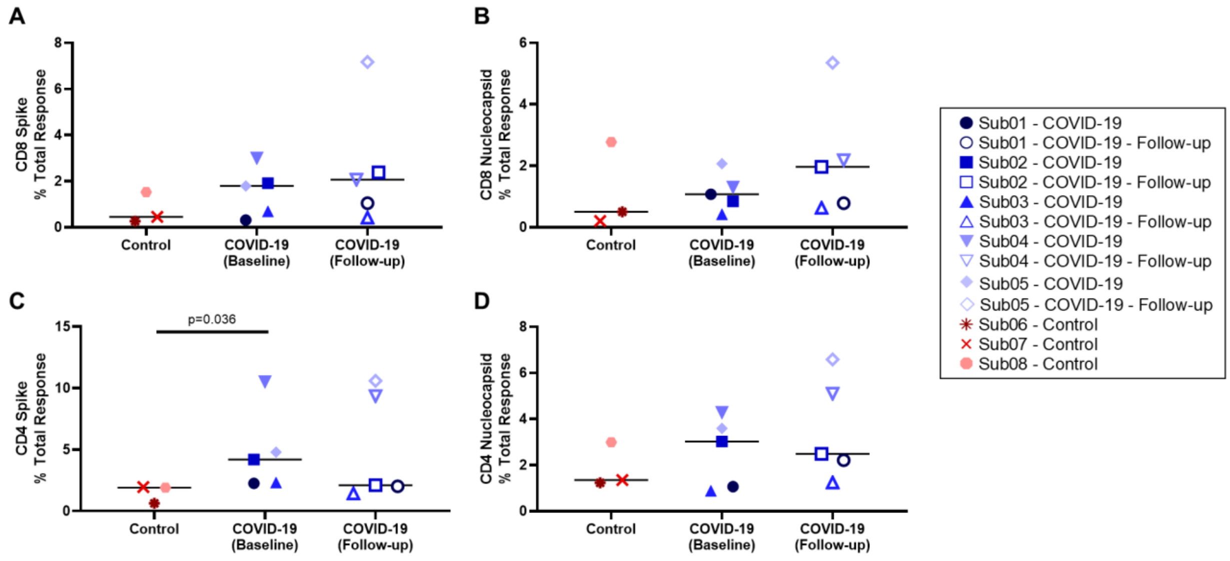

Fig. S16. Total responses in CD8$^+$ and CD4$^+$ memory T cells. Total percentage of (**A** and **B**) CD8$^+$ and (**C** and **D**) CD4$^+$ memory T cells responding in any way (CD107a, IFNγ, IL2, MIP-1β, or TNFα) to SARS-CoV-2 (**A** and **C**) spike and (**B** and b) nucleocapsid proteins compared in peripheral blood of all subjects.

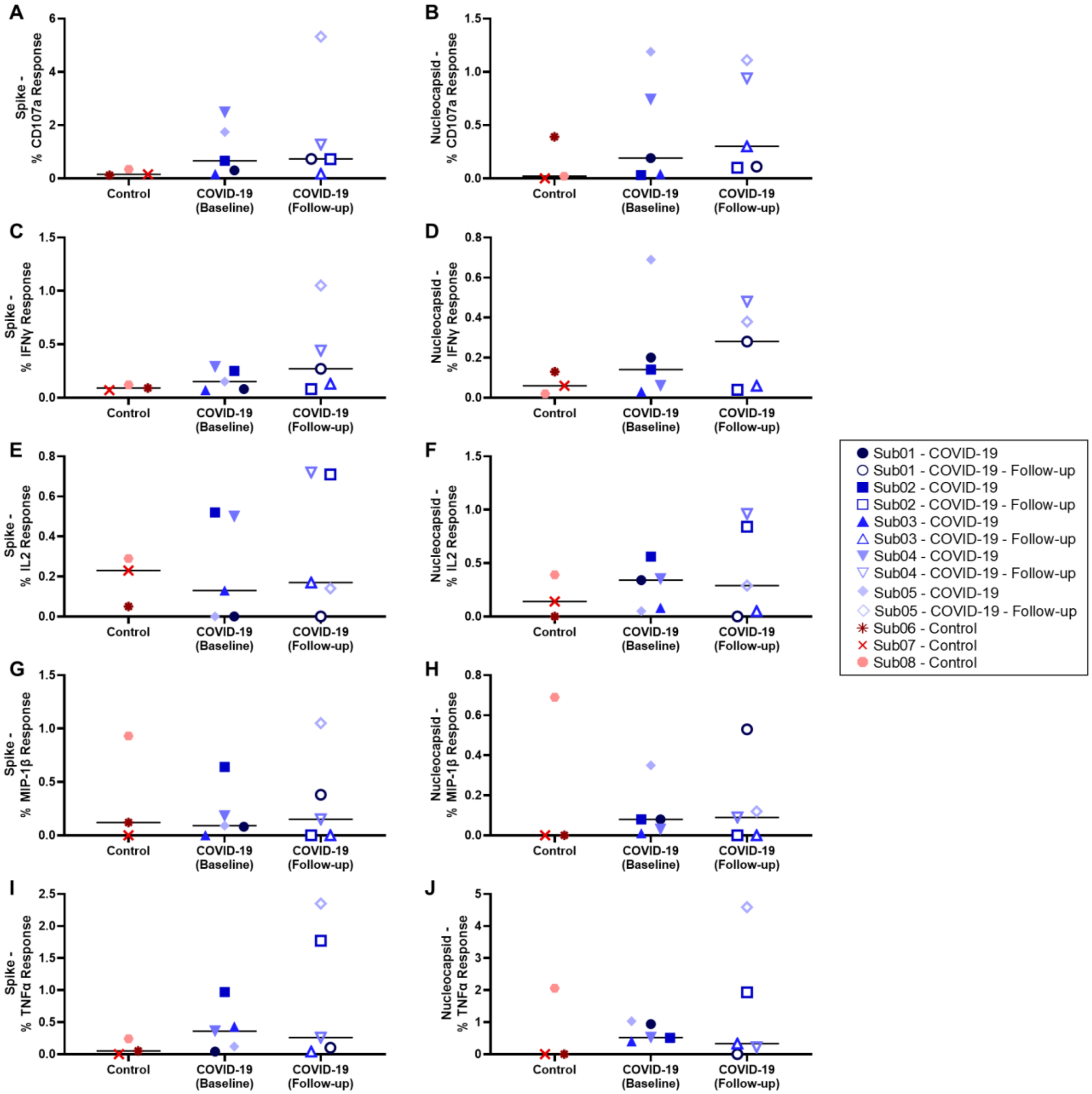

Fig. S17. Memory CD8$^+$ T cell individual functional responses. (**A**, **C**, **E**, **G**, **I**) Percentage of SARS-CoV-2 spike-specific and (**B**, **D**, **F**, **H**, **J**) nucleocapsid-specific CD8$^+$ memory T cells showing (**A** and **B**) CD107a expression, (**C** and **D**) IFNγ production, (**E** and **F**) IL2 production, (**G** and **H**) MIP-1β production, and (**I** and **J**) TNFα production.

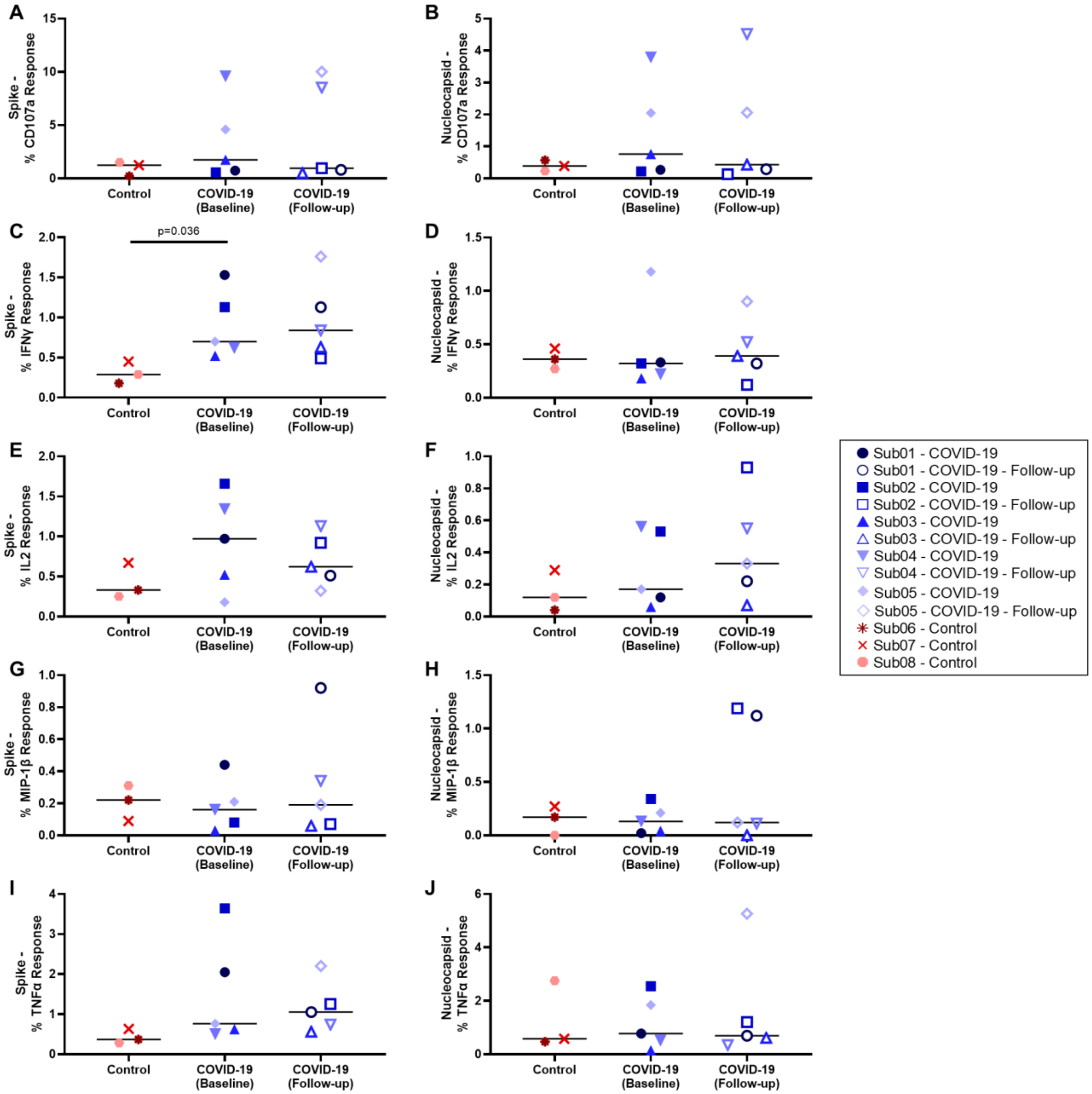

Fig. S18. Memory CD4^{+} T cell individual functional responses. (**A**, **C**, **E**, **G**, **I**) Percentage of SARS-CoV-2 spike-specific and (**B**, **D**, **F**, **H**, **J**) nucleocapsid-specific CD4^{+} memory T cells showing (**A** and **B**) CD107a expression, (**C** and **D**) IFNγ production, (**E** and **F**) IL2 production, (**G** and **H**) MIP-1β production, and (**I** and **J**) TNFα production.

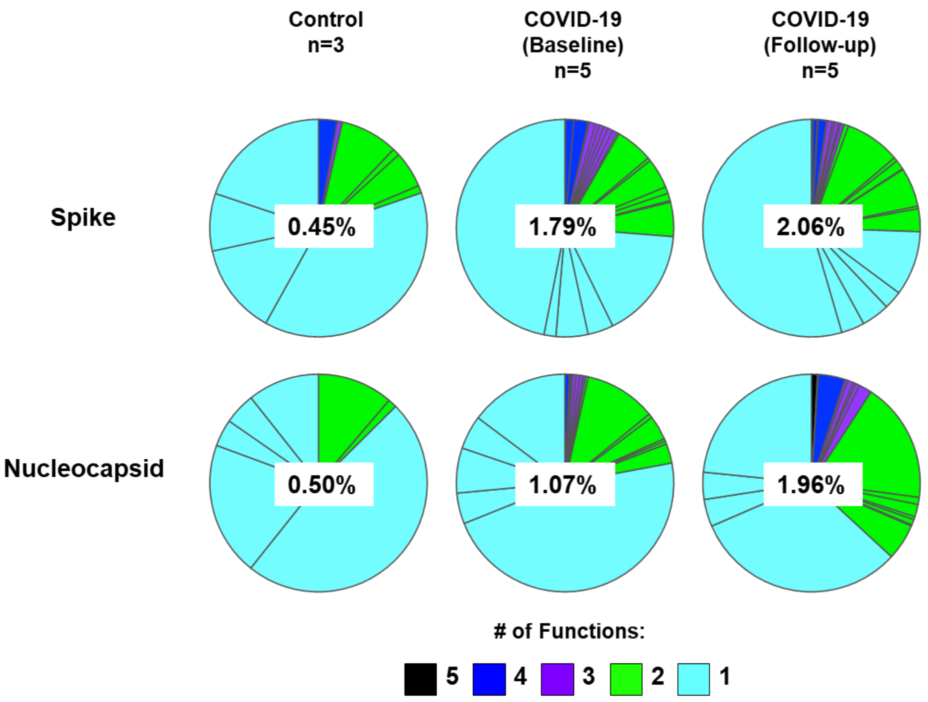

Fig. S19. Polyfunctional responses in SARS-CoV-2 spike-specific and nucleocapsid-specific CD8$^+$ memory T cells. The percentages shown on each pie chart represent the median magnitude of the total response in each group.

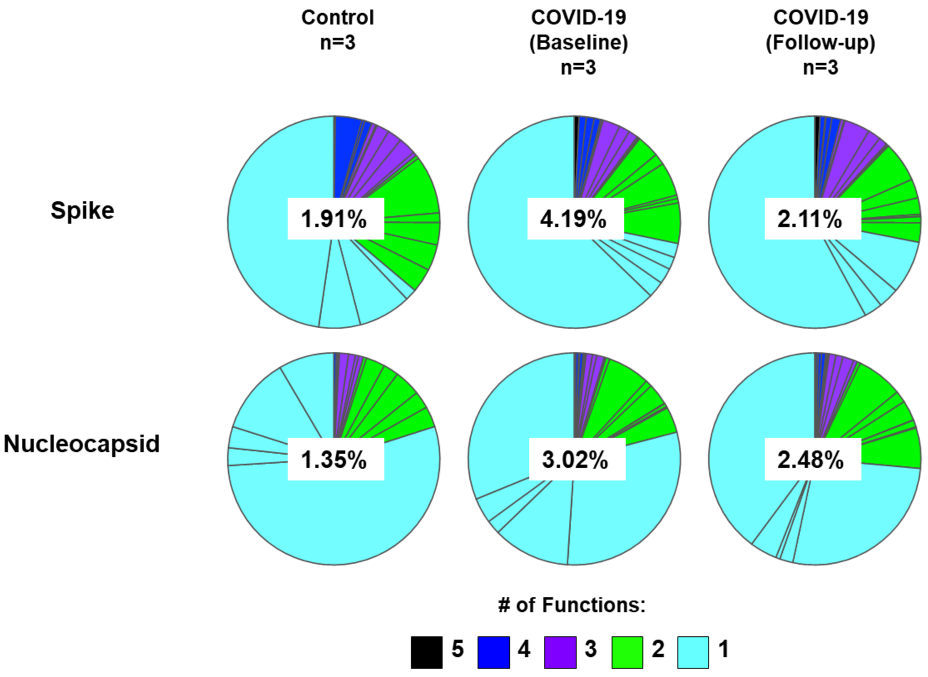

Fig. S20. Polyfunctional responses in SARS-CoV-2 spike-specific and nucleocapsid-specific CD4$^+$ memory T cells. The percentages shown on each pie chart represent the median magnitude of the total response in each group.

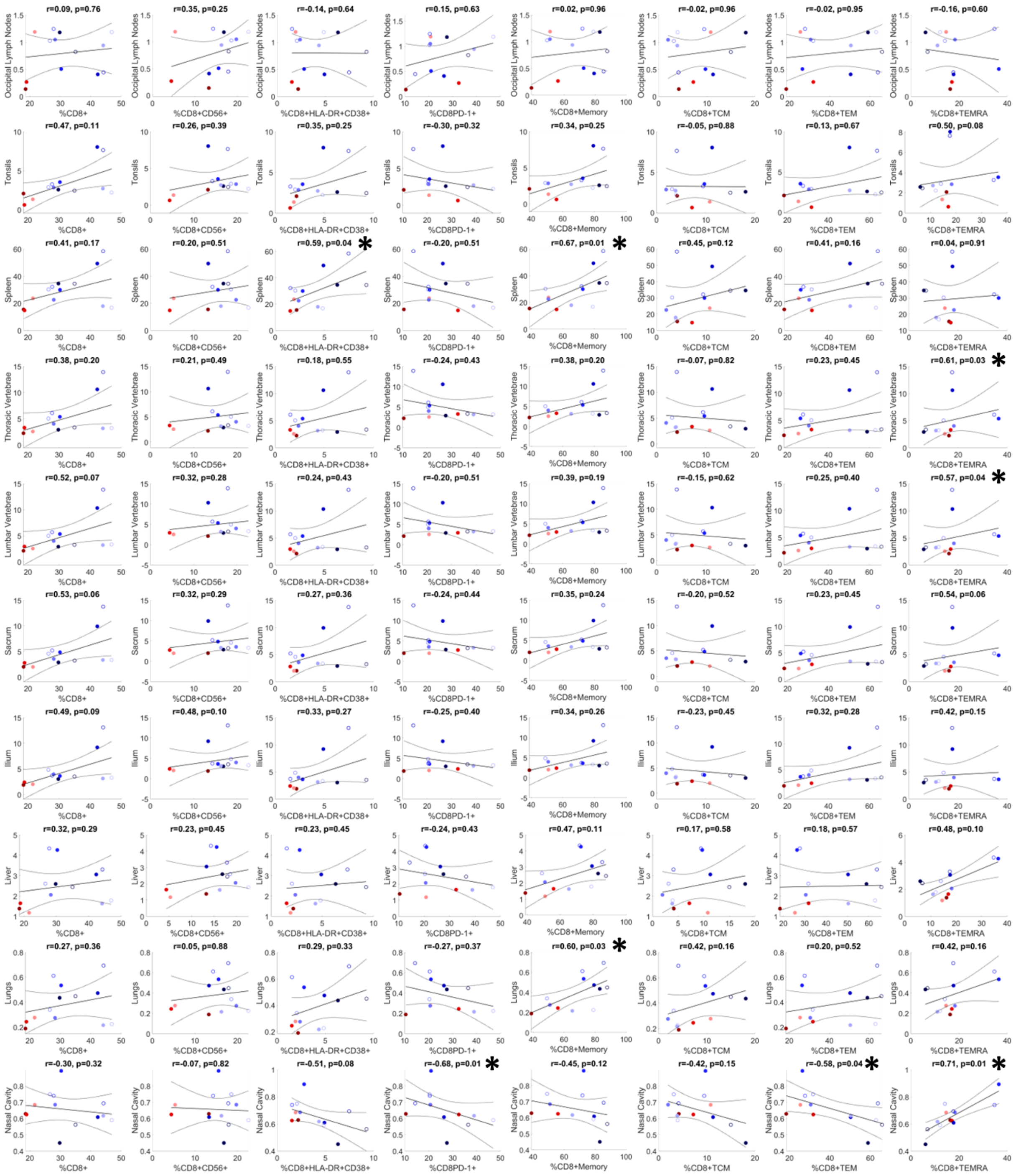

Fig. S 21. Spearman rank correlation analysis between 6-h timepoint TBRs and peripheral blood CD8$^+$ immunophenotypes. Correlations are calculated between TBRs from the 6-h timepoint in occipital lymph nodes, tonsils, spleen, bone marrow (thoracic vertebrae, lumbar vertebrae, sacrum, and ilium), liver, lungs, nasal cavity and peripheral blood CD8$^+$ immunophenotype frequencies, including frequencies of total CD8$^+$ T cells, activated CD8$^+$ T cells (CD56$^+$ and HLA-DR$^+$CD38$^+$), exhausted CD8$^+$ T cells, total CD8$^+$ memory T cells, and subsets of CD8$^+$ memory T cells (T_{CM}, T_{EM}, and TEMRA). *P* values <0.05 are marked with asterisks, not

including any multiple comparison correction. Data points from the control subjects and COVID-19 patients are shown in shades of red and blue, respectively. Solid circles are the baseline scans and the empty circles are the 4-month follow-up scans. Solid lines represent the linear regression fits and the dashed curves represent the 95% confidence bounds.

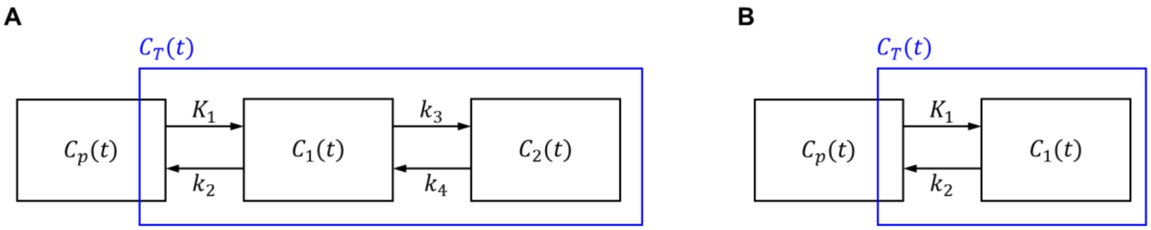

$$C_T(t) = C_{Blood}(t) + C_{Free}(t) + C_{Bound}(t)$$
$$C_{Blood}(t) = v_b C_p(t)$$
$$C_{Free}(t) = (1 - v_b) C_1(t)$$
$$C_{Bound}(t) = (1 - v_b) C_2(t)$$

$$\begin{cases} \dfrac{dC_1(t)}{dt} = K_1 C_p(t) - (k_2 + k_3) C_1(t) + k_4 C_2(t) \\ \dfrac{dC_2(t)}{dt} = K_3 C_1(t) - k_4 C_2(t) \end{cases}$$

$$C_T(t) = v_b C_p(t) + (1 - v_b) C_1(t)$$
$$\frac{dC_1(t)}{dt} = K_1 C_p(t) - k_2 C_1(t)$$

Fig. S22. Definition of conventional one-tissue and two-tissue compartmental models used in PET kinetic modeling. (**A**) Two-tissue compartmental model with five fitting microparameters (2T5P) of ($\boldsymbol{v_b, K_1, k_2, k_3, k_4}$) and (**B**) one-tissue compartmental model with three fitting microparameters (1T3P) of ($\boldsymbol{v_b, K_1, k_2}$), in which $\boldsymbol{C_p}$ corresponds to the activity concentration of the tracer in blood plasma (which is approximated by image-derived whole-blood activity concentration in this study), $\boldsymbol{C_T}$ corresponds to the activity concentration of the tracer in tissue, $\boldsymbol{v_b}$ represents the fractional blood volume in tissue, and $\boldsymbol{K_1, k_2, k_3, \text{and } k_4}$ are the rate constants between the model compartments. Two-tissue compartmental model with fours fitting microparameters (2T4P) can be defined as a special case of 2T5P model, in which $\boldsymbol{k_4}$ is zero. $\boldsymbol{K_1}$ is expressed in mL/min/mL$_{\text{tissue}}$, while $\boldsymbol{k_2, k_3, \text{and } k_4}$ are all expressed in 1/min. The differential equations defining each model are shown below each model. $\boldsymbol{K_1 \text{ and } k_2}$ are affected by the blood flow and the permeability of the tracer across the blood capillaries. In a simplified case, the first compartment represents concentrations of the free tracer in tissue and the second compartment represents concentrations of the tracer bound to cell receptors in the tissue. In the case of 1T3P model, the free and bound compartments are not distinguishable from each other. In the specific case of ^{89}Zr-Df-Crefmirlimab targeting the CD8 receptors, all rate constants include effects from cell trafficking and the concentrations in each compartment also include concentrations of labeled cells trafficking in and out of the tissue.

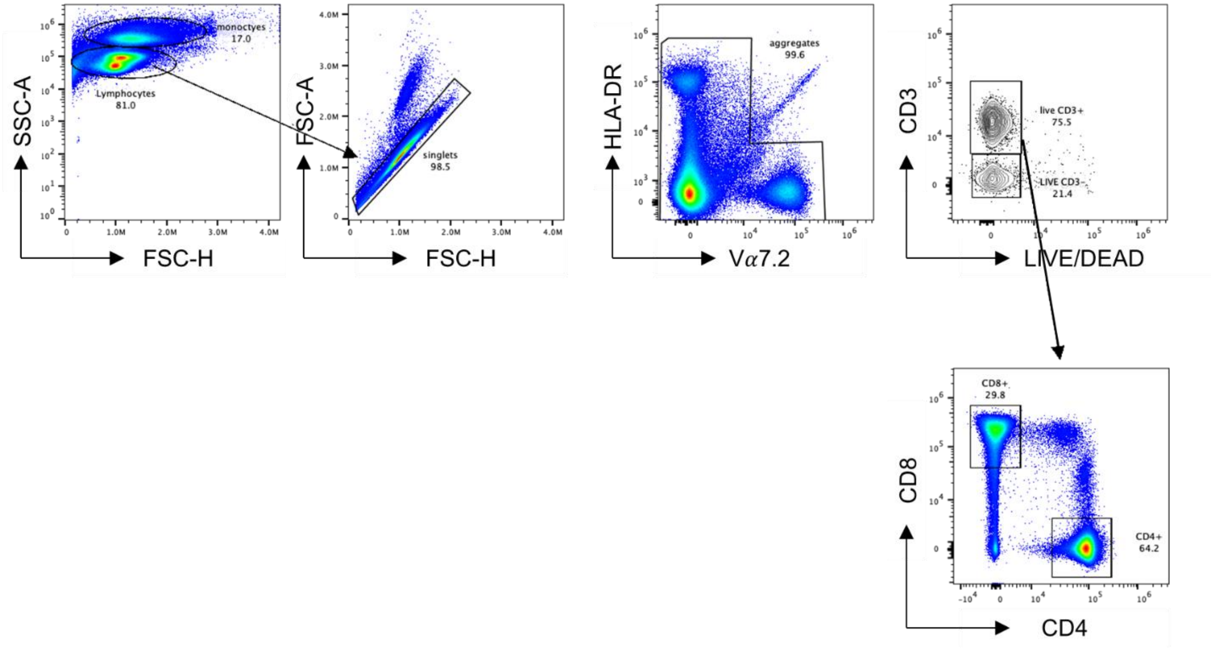

Fig. S23. Gating strategy for T cell immunophenotyping. Single cell lymphocytes were gated, aggregates removed, and stained with LIVE/DEAD cell stain kit to exclude non-viable cells. Viable $CD3^+$ cells were gated as T cells and $CD8^+$ and $CD4^+$ T cells were separated.

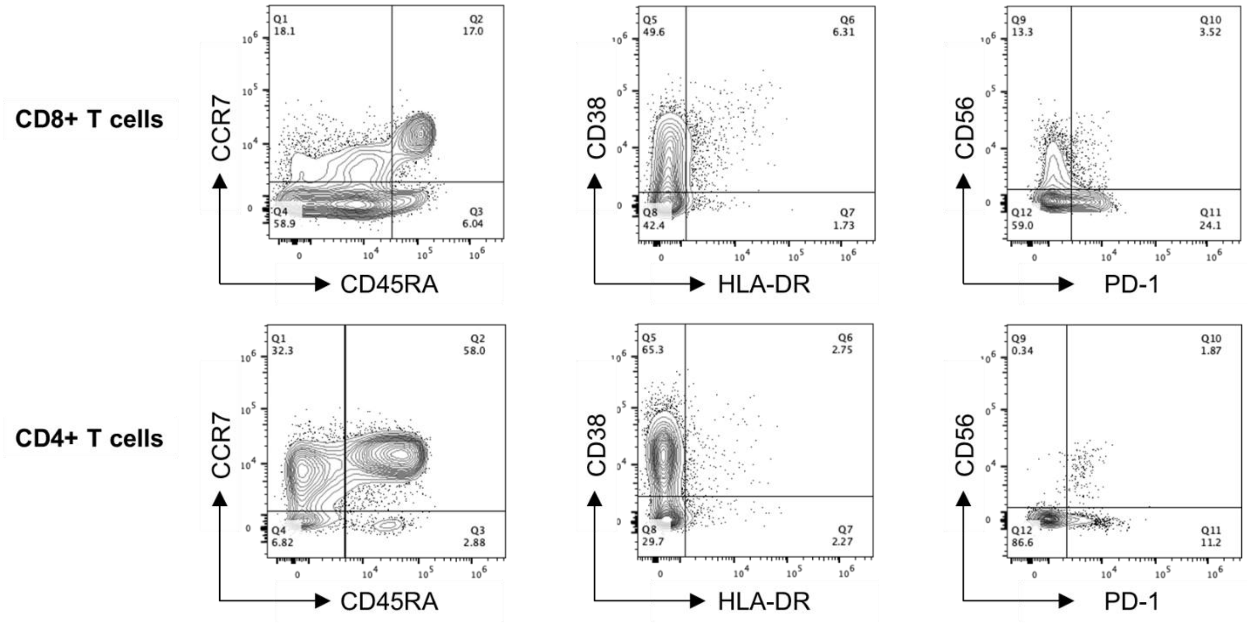

Fig. S24. Gating strategy for memory T cell immunophenotyping. CD45RA and CCR7 were used for gating T cell memory subsets, defined as naïve ($CD45RA^+$, $CCR7^+$), central memory ($CD45RA^-$, $CCR7^+$), effector memory ($CD45RA^-$, $CCR7^-$), and terminally differentiated effector memory ($CD45RA^+$, $CCR7^-$). T cell activation was assessed with co-expression of HLA-DR and CD38, in addition to CD56 expression. Lastly, PD-1 expression was used for quantifying the exhausted T cell population.

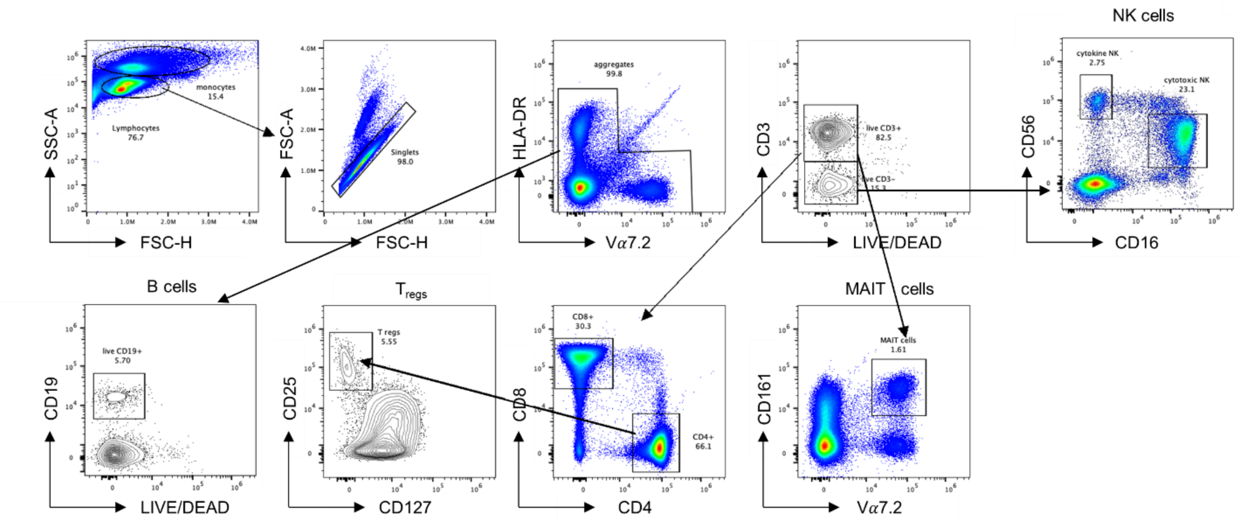

Fig. S25. Gating strategy for phenotyping T$_{reg}$ cells, MAIT cells, NK cells and B cells.

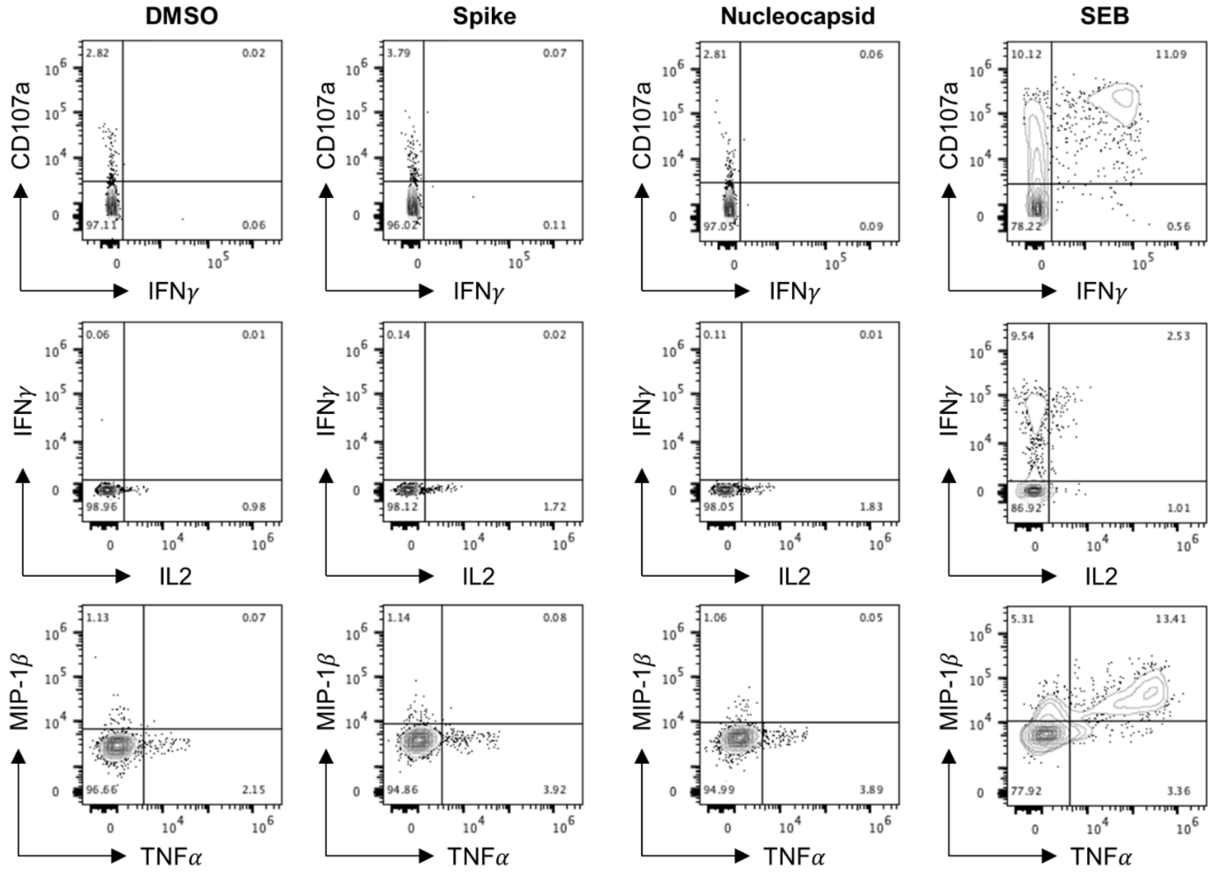

Fig. S26. Gating strategies to define SARS-CoV-2-specific CD8+ memory T cell response, using individual SARS-CoV-2 peptide pools. Representative examples of flow cytometry plots of SARS- CoV-2-specific CD8^{+} memory T cells are shown after overnight stimulation with spike and nucleocapsid peptide pools, compared to negative control (DMSO) and positive control stimulation with SEB.
